# The Impact of Focused Gene Ontology Curation of Specific Mammalian Systems

**DOI:** 10.1371/journal.pone.0027541

**Published:** 2011-12-09

**Authors:** Yasmin Alam-Faruque, Rachael P. Huntley, Varsha K. Khodiyar, Evelyn B. Camon, Emily C. Dimmer, Tony Sawford, Maria J. Martin, Claire O'Donovan, Philippa J. Talmud, Peter Scambler, Rolf Apweiler, Ruth C. Lovering

**Affiliations:** 1 EMBL-European Bioinformatics Institute, Hinxton, Cambridge, United Kingdom; 2 Centre for Cardiovascular Genetics, Institute of Cardiovascular Science, University College London, London, United Kingdom; 3 Molecular Medicine Unit, Institute of Child Health, University College London, London, United Kingdom; University College Dublin, Ireland

## Abstract

The Gene Ontology (GO) resource provides dynamic controlled vocabularies to provide an information-rich resource to aid in the consistent description of the functional attributes and subcellular locations of gene products from all taxonomic groups (www.geneontology.org). System-focused projects, such as the Renal and Cardiovascular GO Annotation Initiatives, aim to provide detailed GO data for proteins implicated in specific organ development and function. Such projects support the rapid evaluation of new experimental data and aid in the generation of novel biological insights to help alleviate human disease. This paper describes the improvement of GO data for renal and cardiovascular research communities and demonstrates that the cardiovascular-focused GO annotations, created over the past three years, have led to an evident improvement of microarray interpretation. The reanalysis of cardiovascular microarray datasets confirms the need to continue to improve the annotation of the human proteome.

**Availability:**

GO annotation data is freely available from: ftp://ftp.geneontology.org/pub/go/gene-associations/

## Introduction

The Gene Ontology (GO) [1], [2] is the most widely used biomedical ontology, with GO terms and gene product annotations displayed by virtually every biological sequence database (including UniProt Knowledgebase, NCBI EntrezGene, GeneCards, Reactome and Ensembl). It is the *de facto* standard for effective analysis of high-throughput datasets. The GO uses structured controlled vocabulary terms, to describe three aspects of a gene product's attributes: the *molecular function(s)*, or activities that a gene product can directly perform; the *biological process(es)* it contributes to; and the subcellular locations (*cellular component*) in which it is active [3]. Over 34,000 GO terms describe a wide range of concepts to differing levels of specificity and are organised as directed acyclic graphs using descriptive relationship types [4]. Full information describing each GO term, such as definitions and synonyms, the associated gene products and publications can be obtained via the QuickGO browser [5].

GO Consortium member groups include a wide range of model organism and database groups who are all involved in the application of automated prediction and/or manual curation methods to generate associations or ‘annotations’ between specific GO terms and genes or gene products for many species [4]. The GO is developed in response to user requests or GO Consortium activities [6], [7]. The four major contributors to the annotation of the human proteome are the UniProt Consortium, the Renal and Cardiovascular GO Annotation Initiatives and the GO Consortium Reference Genome Group [8]–[12].

The renal and cardiovascular research communities have embraced high-throughput technologies to identify, quantify and characterise relevant pathways and networks [13]–[16]. Consequently, the Renal and Cardiovascular Initiatives [10], [11] were instigated to support the interpretation of these datasets by providing a comprehensive public resource of GO annotations for targeted protein sets. The annotation focus of these two initiatives is proteins implicated in renal and cardiovascular development, function and disease. Both initiatives concentrate on improving the ontology describing renal and cardiovascular-associated processes and then use this enriched GO vocabulary to summarize published experimentally validated knowledge for relevant proteins.

Electronic annotation pipelines are invaluable in supplying many millions of valid GO annotations to a wide range of sequences. Applied electronic annotation methods exploit the information available from protein signature [17] or orthology data [18] as well as manual and automated annotation efforts [8]. Each prediction method must generate high-quality annotations, which constrains the number and specificity of their predictions. In contrast, manual annotation requires highly-trained biocurators to read and evaluate evidence from published literature in order to associate appropriate GO terms to proteins [19], [20]. Indisputably, manual annotation is a labor-intensive process, however, it does apply GO terms which are far more informative and accurate than can be achieved by the current electronic pipelines, providing a comprehensive, detailed summary of the published knowledge about a gene product. For example, the human protein WNT7A (www.ebi.ac.uk/QuickGO/GProtein?ac=O00755) has been annotated with the electronic InterPro2GO annotation GO:0007275, ‘*multicellular organismal development*’, however, the manual annotation effort has been able to additionally assign the more descriptive terms GO:0050768, ‘*negative regulation of neurogenesis*’ and GO:0051965, ‘*positive regulation of synaptogenesis*’, to this protein.

There are two general approaches which are used for the manual GO annotation of proteins: the protein-centric approach resulting in comprehensive annotation of a single protein (or protein family) or the process-centric approach in which a biocurator focuses on the annotation of all proteins involved in a single process. The protein-centric approach, has the advantage of identifying a protein's involvement in multiple processes, however it does mean that the biocurator may not appreciate the ‘bigger picture’ relevant to each process, and is more likely to use the available GO terms, rather than request new, more-specific ones. The Renal and Cardiovascular Initiatives mainly utilise the process-centric annotation approach, which leads to detailed curation of groups of similarly functioning proteins. Annotating to a specific process allows the biocurator to gain a more thorough understanding of the role played by each protein within a process, more consistent annotation of these proteins and consequently specific new GO terms tend to be more frequently requested, leading to improvements in a particular GO domain (e.g., the expansion of the plasma lipoprotein particle ontology from one term to eleven terms), or development of an extensive ontology, for example expanding the heart development [21] and kidney development ontologies). Process-focused GO annotation complements the existing GO annotations created by various model organism databases and specialist groups whose annotation sets are not focused on a particular biological area. Together both approaches provide the depth (process-focused approach) and breadth (non-focused approach) of annotations needed for information-rich interpretation of scientific studies.

Both the Renal and Cardiovascular Initiatives have enhanced the quality and quantity of GO terms associated with human proteins. Now that the more established Cardiovascular Initiative has entered its fourth year, we are able to demonstrate how the resulting cardiovascular annotation set can better assist in the interpretation of microarray datasets.

## Results

### Impact of process-focused annotation on the depth of GO annotation

**Figure 1:**
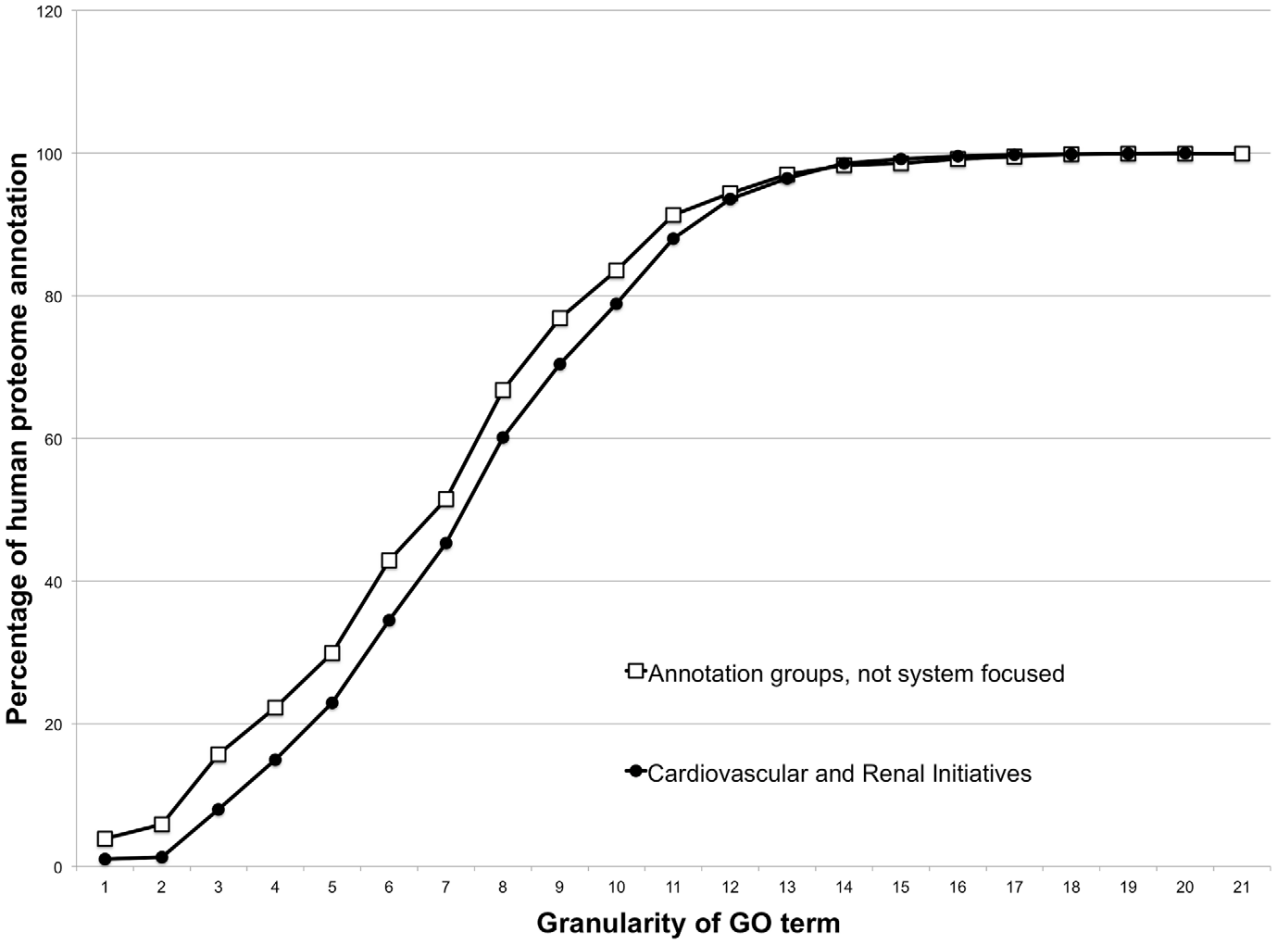
Distribution of GO term specificity by annotation source. Accumulative frequency of the distribution of GO terms applied in human annotations. Manual annotations created by the Cardiovascular and Renal Initiative, compared to those created by annotation groups without a system focused approach. Mann Whitney U confirms that the median granularity of GO terms used in human protein annotation by the Cardiovascular and Renal Initiatives is 8.0 (inter quartile range 6–10), compared to a median granularity of 7.0 (inter quartile range 5–9), for the GO terms used by other groups manually annotating to the human proteome (P<0.0001).

Improvements to protein annotation were measured by comparing the number and specificity of annotations supplied to human proteins by the Renal and Cardiovascular Initiatives with those supplied by other groups to the human proteome. The Cardiovascular and Renal Initiatives have increased the average number of GO annotations/protein for their prioritised protein lists, compared to the average number of GO annotations/protein in the human proteome. For example, on 11^th^ July 2011 the human proteome was manually annotated with an average of 10 GO terms per protein (15,866 proteins), whereas, the 4,500 human proteins prioritised for annotation by the Renal and Cardiovascular Initiatives have an average of 16 manual annotations per protein. Improvements to the human GO annotation set were also measured by comparing the specificity of annotations supplied to human proteins by the Renal and Cardiovascular Initiatives with those supplied by other groups. This comparison demonstrated that the annotations contributed by the system focused annotation approaches supply high information content, indicated by the increase in the specificity of the terms applied (defined in terms of granularity, Figure 1). Performing the Mann Whitney U test on this data confirms that the median granularity of GO terms used in human protein annotation by both the Cardiovascular and Renal Initiatives is 8.0 (inter quartile range 6–10), compared to a median granularity of 7.0 (inter quartile range 5–9), for the GO terms used by other groups manually annotating to the human proteome (P<0.0001).

### Impact of process-focused annotation on ontology development

**Figure 2:**
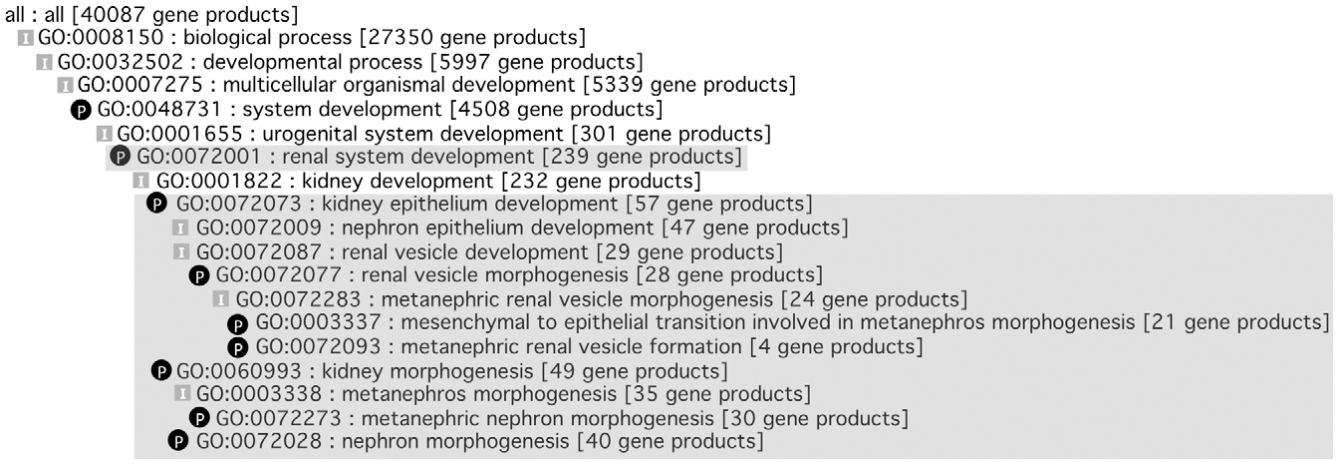
AmiGO ‘Tree View’ image of part of the kidney developmental process ontology. The ‘tree view’ in AmiGO (http://amigo.geneontology.org) showing the GO term parents of GO:0003337 ‘*mesenchymal to epithelial transition involved in metanephros morphogenesis*’. The most specific twelve GO terms (shaded) were amongst the 470 new terms created following the kidney development ontology workshop. The numbers in brackets indicate the number of **human** proteins annotated to the GO term, or one of its child terms (07^th^ October 2011). [I] *is_a* parent-child relationship, ‘P’ *part_of* parent-child relationship.

At the start of the Renal and Cardiovascular GO initiatives, only 12 terms for heart development and 22 terms for kidney development were available in GO to cover the complex processes involved in the development, specification and differentiation of these organs and highly differentiated tissue specific cells. Therefore, to achieve improvements in these areas, both curator and workshop-led ontology development activities were instigated. Cross-species collaborations ensured that organism anatomy was correctly applied by the new GO terms and facilitated species-neutral ontology development, which supports the transfer of annotations from characterised to poorly-studied, closely-related species. These activities generated 283 cardiovascular terms [21] and 479 renal terms, and a small section of the improved GO for kidney development terms, with associated annotations, is shown (Figure 2; a search at AmiGO http://amigo.geneontology.org with GOC:mtg_heart or GOC:mtg_kidney gives a full list of these new GO terms). Improving the ontology enables more specific gene groups to be created, For example, using the new kidney terms in the ontology, 24 human proteins are now annotated with the informative biological process GO term ‘*metanephric renal vesicle morphogenesis*’, rather than the only appropriate GO term previously available ‘*kidney development*’, which is associated with over 200 human proteins. In comparison, there are currently only 34 terms describing the biological processes involved in eye development, demonstrating that the ontologies relevant to complex organ systems without focused annotation efforts are not being prioritised for ontology development.

### Impact of process-focused annotation on high-throughput data analysis

The Cardiovascular Initiative began in 2007 and the manual annotations added since then have led to the generation of a large GO annotation dataset. This set was therefore chosen to examine the possible impact on the analysis of two separate high-throughput datasets, as described below.

#### Analysis 1

A microarray dataset was chosen for reanalysis that had examined differentially regulated genes in peripheral blood mononuclear cells from patients with systemic scleroderma-related pulmonary arterial hypertension (PAH-SSc) compared to healthy controls [22]. The original interpretation of this microarray dataset was achieved using GenMAPP and MAPPFinder which identified 9 GO terms as enriched in this dataset, including ‘*angiogenesis*’, ‘*chemotaxis*’ and ‘*inflammatory response*’ (see Table 1). Without the raw data we were unable to use the MAPPFinder tool used by Grigoryev *et al*., instead we used three different GO term enrichment tools to look at how data interpretation changes with the addition of new GO annotations. The analysis tools GO-Elite, the sister program of MAPPFinder [23], which was expected to produce similar results to MAPPFinder, Ontologizer [24] and ProfCom GO [25]. The latter two tools were chosen as they were widely-respected, popular tools that facilitated the inclusion of specified and filtered GO annotation datasets.

**Table 1:** Comparison of PAH-SSc microarray data analysis using MAPPFinder in 2008 and GO-Elite, Ontologizer and ProfCom GO in 2011.

	GenMAPP analysisGrigoryev *et al*, 2008			GenMAPP GO-Elite analysis June 2011			Ontologizer analysisMarch 2011			ProfCom GO analysisMarch 2011		
GO term	Z-score	S	P	Z-score	St = 262	Pt = 17158	p-value (Adj)	St = 264	Pt = 18249	p-value	St = 265	P t = 18257
angiogenesis	**5.534**	6	41	**6.3**	11	137	1	15	248	8.00E-02	9	122
chemotaxis	**6.457**	10	111	**4.75**	20	484	1	20	525	7.50E-02	9	121
inflammatory response	**8.429**	18	179	**6.6**	17	265	1	23	361	**2.03E-04**	16	224
cellular component movement	**4.378**	11	108	**7.5**	28	506	**0**	35	701	9.90E-02	8	98
G-protein coupled receptor signaling	**5.576**	15	825	**3.66**	18	524	1	19	573	N/A	N/A	N/A
cell-cell signaling	**3.208**	11	283	−0.36	8	597	1	15	877	N/A	N/A	N/A
sensory perception	**2.323**	7	472	N/A	N/A	N/A	1	3	835	N/A	N/A	N/A
antimicrobial humoral response	**2.706**	6	84	N/A	N/A	N/A	N/A	N/A	N/A	N/A	N/A	N/A
Negative regulation of cell proliferation	**3.383**	9	136	4.07	16	403	1	16	399	N/A	N/A	N/A

Significant Z-scores and p-values are highlighted in bold text. GO processes with Z scores >1.96 identified by MAPPFinder and GO-Elite are considered as significantly enriched [22]; adjusted p-values<0.1 identified by Ontologizer [24] are considered as significantly enriched; P-values.<0.01 identified by ProfCom GO [25] are considered as significantly enriched. S  =  study count, P  =  population count, t  =  number of protein IDs.

**Figure 3:**
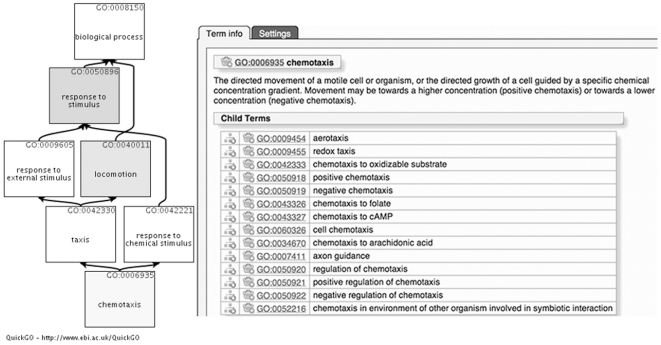
QuickGO term display. QuickGO (www.ebi.ac.uk/QuickGO) ancestor chart showing information for GO:0006935 ‘*chemotaxis*’ and its ‘*is_a*’ parent relationships within the hierarchical directed acyclic graph. The GO terms ‘*chemotaxis*’, ‘*locomotion*’ and ‘*response to stimulus*’ are highlighted to illustrate their parent-child relationships. The child term details are displayed for the GO term ‘*chemotaxis*’.

Re-analysis of the original Grigoryev *et al*. dataset with GO-Elite (Table S1) confirmed that the majority of the GO terms identified by Grigoryev *et al*. were also significantly enriched using the 2011 dataset. However, 3 terms, ‘*cell-cell signalling*’, ‘*sensory perception*’ and ‘*antimicrobial humoral response*’ described by Grigoryev *et al*. as being enriched were no longer identified. In contrast to this we found only one GO term significantly enriched in the Ontologizer analysis and one term in the ProfCom GO analysis that were also reported as enriched by Grigoryev *et al*. (‘*cellular component movement*’ and ‘*inflammatory response*’ respectively, see Table 1). However, GO terms closely related to several of the terms identified by Grigoryev *et al*. were enriched using Ontologizer and ProfCom. For example, the Ontologizer analysis identifies the GO terms ‘*response to stimulus*’ and ‘*locomotion*’, and these terms are parents to the ‘*chemotaxis*’ term identified by Grigoryev *et al*. (see Figure 3 and Table 2). The ProfCom GO analysis also enriched for the ‘*chemotaxis*’-related term ‘*leukocyte migration*’, and the ‘*antimicrobial humoral response*’-related term ‘*response to lipopolysaccharide*’ (Table 3).

**Table 2:** Comparison of Ontologizer PAH-SSc microarray data analysis using GO annotation dataset with and without the human protein annotations submitted by the Cardiovascular Initiative.

		GO dataset including Cardiovascular Initiative annotations				GO dataset without Cardiovascular Initiative annotations			
GO ID	GO term	p-value	p-value (Adj)	Study count(t = 264)	Population count(t = 18249)	p-value	p-value (Adj)	Study count(t = 264)	Population count(t = 18244)
GO:0002376	immune system process	1.38E-20	**0**	77	1487	2.73E-18	**0**	71	1406
GO:0065007	biological regulation	1.15E-10	**0**	183	8119	3.02E-10	**0**	179	7943
GO:0050896	Response to stimulus	2.93E-10	**0**	155	6423	1.57E-09	**0**	151	6318
GO:0040011	Locomotion	8.55E-09	**0**	42	970	4.88E-07	**0**	36	883
GO:0016265	Death	1.56E-08	**0**	53	1431	6.33E-09	**0**	52	1354
GO:0023052	Signaling	1.76E-08	**0**	107	4017	6.16E-09	**0**	106	3898
GO:0006928	cellular component movement	6.01E-08	**0**	35	701	9.27E-07	**0**	30	612
GO:0032502	developmental process	1.15E-07	**0**	98	3678	1.67E-07	**0**	95	3553
GO:0001775	cell activation	1.73E-07	**0.002**	32	632	7.56E-06	**0**	27	575
GO:0006950	Response to stress	4.68E-07	**0.002**	92	2552	1.57E-05	**0.004**	84	2448
GO:0008283	cell proliferation	3.15E-06	**0.002**	42	1205	2.37E-05	**0.006**	37	1091
GO:0009987	cellular process	1.27E-05	**0.004**	229	12453	7.36E-06	**0**	228	12356
GO:0032501	multicellular organismal process	3.01E-05	**0.008**	117	5194	3.81E-05	**0.01**	114	5054
GO:0048518	positive regulation of biological process	3.91E-05	**0.01**	87	2786	1.60E-04	**0.05**	78	2541
GO:0022610	biological adhesion	4.59E-05	**0.01**	30	827	5.74E-04	0.186	26	782
GO:0009605	Response to external stimulus	6.49E-05	**0.014**	44	1033	1.66E-04	**0.05**	40	952
GO:0001816	Cytokine production	6.75E-05	**0.014**	18	289	1.64E-03	0.458	13	227
GO:0048519	Negative regulation of biological process	7.13E-05	**0.016**	77	2404	4.42E-04	0.144	68	2190
GO:0051674	localization of cell	7.73E-05	**0.016**	29	606	1.27E-03	0.37	23	515
GO:0051179	Localization	1.14E-04	**0.038**	87	3669	2.48E-04	**0.078**	82	3482
GO:0065008	regulation of biological quality	1.80E-04	**0.06**	72	2199	2.39E-04	**0.076**	67	2024
GO:0042221	Response to chemical stimulus	2.13E-04	**0.076**	74	2177	1.25E-03	0.364	67	2048
GO:0046209	nitric oxide metabolic process	2.41E-04	**0.094**	6	51	2.88E-02	1	3	40
GO:0003013	circulatory system process	9.57E-04	**0.318**	12	263	2.16E-04	**0.068**	12	226

Significant adjusted p-values are highlighted in bold text. GO processes with adjusted p-values<0.1 identified by Ontologizer [24] are considered as significantly enriched. t  =  number of protein IDs.

**Table 3:** Comparison of ProfCom GO PAH-SSc microarray data analysis using GO annotation dataset with and without the human protein annotations submitted by the Cardiovascular Initiative.

		GO dataset including Cardiovascular Initiative annotations			GO dataset without Cardiovascular Initiative annotations		
GO ID	GO term	p-value	Study count(t = 265)	Population count(t = 18257)	p-value	Study count(t = 265)	Population count(t = 18252)
GO:0032496	response to lipopolysaccharide	**5.45E-05**	12	107	**1.22E-03**	10	95
GO:0006954	inflammatory response	**2.03E-04**	16	224	1.59E-02	13	214
GO:0045768	positive regulation of anti-apoptosis	**5.28E-04**	7	34	**2.06E-03**	6	27
GO:0045429	postive regulation of nitric oxide biosynthetic process	**1.75E-03**	6	26	#N/A	#N/A	#N/A
GO:0006955	immune response	**1.82E-03**	23	510	**1.30E-03**	23	502
GO:0048661	positive regulation of smooth muscle cell proliferation	**2.22E-03**	6	27	**6.23E-03**	5	19
GO:0014070	response to organic cyclic substance	**3.02E-03**	10	104	**2.82E-03**	10	104
GO:0050900	leukocyte migration	**3.60E-03**	10	106	1.94E-02	9	103
GO:0051412	response to corticosterone stimulus	**6.69E-03**	5	19	**6.23E-03**	5	19
GO:0019221	cytokine-mediated signaling pathway	**9.66E-03**	13	203	1.32E-01	11	194

Significant p-values are highlighted in bold text. GO processes with p-values<0.01 identified by ProfCom GO [25] are considered as significantly enriched. t  =  number of protein IDs.

The re-analysis of the Grigoryev *et al*. dataset using the more recent GO annotation datasets identified the significant enrichment of additional GO terms, which were not originally reported. In total the GO-Elite analysis identified 696 significantly enriched ‘*biological process*’ GO terms, Ontologizer 24 GO terms, and ProfCom GO 10 GO terms, many of which are relevant to the PAH-SSc phenotype such as ‘*response to cytokine stimulus*’ , ‘*response to organic cyclic substance*’ and ‘*regulation of NF-kappaB import into nucleus*’ (Tables S1, 2 and 3). In order to fully examine the impact of the Cardiovascular Initiative on the analysis of the PAH-SSc dataset, the term enrichment was repeated using the March 2011 GO annotation dataset from which all of the Cardiovascular Initiative submitted annotations had been removed (13,000 annotations). Unfortunately, only the Ontologizer and ProfCom GO tools provided the facility to input these filtered datasets. Removing the Cardiovascular Initiative annotations decreased the significance of the majority of the enriched GO terms, and 6 GO terms were no longer significantly enriched (Table 2). Several of these 6 GO terms are relevant to the disease phenotype; e.g. ‘*cytokine production*’ and ‘*nitric oxide metabolic process*’ (Table 2), confirming an improved interpretation of the dataset with the annotations supplied by the Cardiovascular Initiative. A difference in the analysis of the PAH-SSc dataset was also seen using ProfCom GO and the full GO annotation dataset compared to the filtered annotation dataset (Table 3), with relevant terms such as ‘*positive regulation of nitric oxide biosynthetic process*’ and ‘*cytokine-mediated signalling pathway*’ only significantly enriched with the inclusion of the Cardiovascular Initiative annotations.

#### Analysis 2

A macrophage microarray dataset was chosen as macrophages play a key role in atherosclerosis and because proteins associated with immune system processes have not previously been targeted for GO annotation. The microarray dataset contained 342 mouse genes differentially expressed in resolution-phase macrophage verses naïve and inflammatory macrophages [26]. Resolution-phase macrophages are a newly identified class of macrophage, with a hybrid phenotype between the alternatively and classically activated macrophage classes [27]. After full annotation of 37 of these genes we used Ontologizer to analyse the full differentially expressed dataset using the mouse GO annotation dataset available before our targeted annotation of a subset of these genes (December 2010) and compared this to the analysis of the same microarray dataset using a later version of the mouse GO annotation dataset (April 2011), which would have also included annotations created during this time by Mouse Genome Informatics. GO term enrichment of this macrophage microarray dataset using the December 2010 GO dataset identified 2 significantly enriched ‘*biological process*’ GO terms: ‘*cell activation*’ and ‘*immune system process*’ (Table 4). The reanalysis using the more recent GO annotation dataset (April 2011) substantially improved the interpretation of the dataset, not only identifying an additional 7 significantly enriched GO terms, but also enriching for GO terms which suggest an involvement of resolution-phase macrophages in stimulating ‘*leukocyte apoptosis*’, ‘*cytokine production*’ and ‘*cell proliferation*’ [26].

**Table 4:** Comparison of Ontologizer macrophage data analysis using GO annotation datasets from December 2010 and April 2011.

		April 2011			December 2010		
GO ID	GO term	p-value (Adj)	Study count(t = 257)	Population count(t = 14241)	p-value (Adj)	Study count(t = 258)	Population count(t = 14386)
GO:0001775	cell activation	**0**	25	390	**0.01**	19	363
GO:0002376	immune system process	**0**	39	885	**0.016**	31	833
GO:0008283	cell proliferation	**0**	38	862	0.236	27	800
GO:0001816	cytokine production	**0.002**	16	220	1	8	205
GO:0042221	response to chemical stimulus	**0.028**	55	1538	1	37	1356
GO:0006928	cellular component movement	**0.038**	24	531	1	12	483
GO:0051674	localization of cell	**0.066**	23	507	1	11	457
GO:0032502	developmental process	**0.068**	81	3006	0.982	65	2832
GO:0071887	leukocyte apoptosis	**0.092**	7	41	1	2	24

Significant p-values are highlighted in bold text. GO processes with adjusted p-values<0.1 identified by Ontologizer [24] are considered as significantly enriched. t  =  number of protein IDs.

## Discussion

The interpretation of large-scale genetic, genomic and proteomic studies depends on computational analyses that incorporate functional annotations. The large number of publications from microarray and proteomics investigations which evaluate the involvement of large sets of genes or proteins in a particular process or response demonstrate that ontological resources, such as Gene Ontology, are routinely used to inform results.

There is a multitude of freely available GO term enrichment tools to use for the interpretation of high-throughput datasets. These tools apply different analysis methods, statistics, multiple correction methods, filters and versions of the GO Consortium ontology and annotation files to analyse gene or protein lists [28]. In this paper, we compare the analysis of a single microarray dataset using four different term enrichment tools, each of which provide different, but often overlapping, data interpretation.

The Ontologizer Parent-Child Intersection analysis takes into account relationships within the GO hierarchy [24] and identifies 24 GO terms as enriched in the PAH-SSc dataset (Table 2). This approach avoids false positives by only regarding a parent term as significant and not any of its child terms, which may have also been over-represented in the annotation set. In contrast, ProfCom GO uses the computationally efficient greedy heuristics algorithm, which identifies the best local solution while searching the global optimum [25] and identifies only 10 significantly enriched GO terms (Table 3). While GO-Elite uses a term-for-term approach, giving a standardised difference (Z-score) based on a hypergeometric distribution [23], and hence finds 700 GO terms that are significantly enriched in this dataset (Table S1).

Such variability in the number of GO terms enriched in each of these analysis tools brings into question the robustness of the GO term enrichment approach, however, there is some consistency in the results obtained and an enrichment of several ‘*biological processes*’ with known roles in pulmonary arterial hypertension (PAH) and scleroderma, with each of the tools. For example, vascular remodeling plays an important role in PAH [29] and GO terms relating to this process are enriched using all of the four tools, ‘*angiogenesis*’ (MAPPFinder and GO-Elite), ‘*positive regulation of smooth muscle cell proliferation*’ (ProfCom GO and GO-Elite), ‘*nitric oxide metabolic process*’ (Ontologizer) and ‘*positive regulation of nitric oxide biosynthetic process*’ (ProfCom GO and GO-Elite, Tables 1, 2, 3 and S1). In addition, systemic scleroderma is an autoimmune disease [22] and the consistent significant enrichment of ‘*cytokine production*’ (Ontologizer and GO-Elite), ‘*cytokine-mediated signaling pathway*’ (ProfCom GO and GO-Elite), and ‘*inflammatory response*’ (MAPPFinder, ProfCom GO and GO-Elite) reflects the inflammatory aspect to this disease. The comparison of GO terms enriched using these four tools therefore confirms that despite the different outputs there is some reproducibility in the interpretation of this dataset. Some specific GO terms identified in only one or two of the analyses also are consistent with the inflammatory nature of the disease, such as ‘*regulation of NF-kappaB import into nucleus*’ and ‘*leukocyte migration*’, and also reflect the treatment of the disease, for example ‘*response to organic cyclic substance*’, all of the PAH-SSc patients were on medication, the majority of which were organic cyclic compounds [22].

A comparison of the significantly enriched GO terms identified using the Cardiovascular Initiative inclusive GO annotation dataset against those enriched without these annotations, confirmed that annotations created by the Cardiovascular Initiative have improved the analysis of the PAH-SSc dataset, using both Ontologizer and ProfCom GO (Tables 2 and 3). Hence, our analyses confirm that GO annotations created through three years of annotation focused on cardiovascular-relevant proteins, rather than specific annotation of just a few genes within a study dataset, has led to a significantly improved interpretation of this PAH-SSc dataset.

Similarly, the comprehensive GO annotation of only 37 of the 342 mouse genes differentially expressed in resolving macrophage versus naïve and inflammatory macrophages, demonstrates that improved annotation of even a small number of process-specific proteins can result in significant enrichment of relevant GO terms in the analysis of a specific large-scale proteomic or genomic dataset (Table 4).

### Conclusion

The Cardiovascular and Renal GO Annotation Initiative approaches have been able to supply high-quality, detailed annotations and specific GO terms. A limited analysis of this data, through reanalysis of GO term enrichment results of the human PAH-SSc and mouse macrophage datasets demonstrate the impact that these focused annotation efforts can have on the interpretation of high-throughput datasets. These results also confirm the need for comprehensive, information-rich annotation datasets and a more knowledgeable use of existing public data to aid in pathway identification and to fully harness bioresources and biomodelling. Hence the continued improvements in both protein GO annotation and ontology development can enable researchers to gain improved biological insights into their proteins of interest and hence guide their future research towards alleviating various human diseases.

Although the Renal and Cardiovascular Initiatives' curators have focused on the annotation of a limited number of proteins, these projects aim to annotate a wide range of functions and processes, not just those associated with renal and cardiovascular processes. However, the production of a process bias in the human annotation dataset is a possible side effect of this approach, which could impact on the analysis of high-throughput datasets. As yet, we have found no evidence of unexpected cardiovascular and renal terms, being detected in term enrichment analyses. Recent microarray analysis, using GO, of vulvar carcinoma [30] and H5N1 influenza infected lungs [31] mostly identified enrichment of only general biological process terms, such as ‘*cell death*’, ‘*cell growth*’, ‘*cell communication*’ and ‘*cell-to-cell signaling*’. Although, the lung analysis also identified enrichment of more specific GO terms, such as ‘*viral reproduction*’, ‘*chemotaxis*’ and ‘*vesicle-mediated transport*’. As the Cardiovascular Initiative has identified over a fifth of the human proteome (4,000 proteins) as relevant to cardiovascular processes, and with the number of renal protein targets increasing (and currently standing at over 1,300), neither of these annotation projects should be considered as narrowly focused efforts. However, this concern does highlight the importance of annotation providers being able to instigate complementary annotation efforts to enhance annotations and terms across a diverse set of proteins. For example, future projects could prioritize the focused annotation of all the subunits in a specific subcellular component, or protein families with similar catalytic activities across closely-related organisms.

Lack of annotation data can lead to investigators to focus only on genes they recognise [32], [33] or to manually annotate genes in their own study groups [22], [33], [34]. These types of approaches can potentially bias data integration and result in valuable targets being over-looked. However, as targeted manual annotation appears to be becoming standard practice, we have demonstrated, through the comprehensive annotation of a few proteins within a microarray dataset, that focused annotation can have a significant impact on data interpretation (Table 4).

Understanding the variability in the annotation of the human proteome should enable users to interpret their analyses in a more critical manner. As with all term enrichment analyses, care must always be taken when interpreting some of the identified GO terms, and users need to consider whether enrichment of the more general parent terms is more physiologically relevant, or whether the term provides a meaningful interpretation of the data at all. For example, ‘*biological regulation*’, ‘*negative regulation of biological process*’ and ‘*cellular process*’ are high-level, (i.e. non-specific) GO terms that convey little information about the exact role of a gene product in a specific process. These types of terms appear quite regularly in GO term enrichment analyses as a large percentage of gene products will be involved in one or several cellular processes, or regulation thereof, but it does not add any value to the interpretation of a dataset to regard these non-specific terms as important. The continuing development of existing (and new) tools and the lack of information in published papers about the source of the annotation datasets, the ontology and tool versions and the statistical methods used in an analysis make it impossible to precisely reproduce the analysis of a dataset. Full disclosure of the datasets and methods needs to become standard practice to enable the interpretation of high-throughput datasets to be reproducible and accountable. Our multiple analyses have confirmed that despite considerable variation in the number of GO terms enriched, many of the key processes, which would be expected to be associated with PAH-SSc disease phenotype, are significantly over-represented in each of the output files from a variety of different tools. This demonstrates a current need to use appropriate, as well as a variety, of term enrichment tools for the evaluation of a high-throughput dataset, to ensure a balanced and reproducible interpretation (for information about the choice of term enrichment tools see Rhee *et al*. [35]).

The impact of both the Renal and Cardiovascular Initiatives on renal and cardiovascular research can be greatly improved through the involvement of experts from the respective research communities and model organism databases. Consequently, a range of online facilities have been made available to encourage scientists to review and comment on GO annotations, suggest improvements to the descriptiveness of renal and cardiovascular-related GO terms and to suggest publications or proteins for curation (available at www.ebi.ac.uk/GOA/contactus.html and www.ucl.ac.uk/cardiovasculargeneontology/feedback). In this way it is possible to ensure that current accumulated knowledge has been comprehensively reviewed and correctly summarized by the dedicated curation team. Members of these communities have already participated in these initiatives and have contributed to the consistent representation of a variety of processes across a range of species.

Any biological community group who would be interested in supporting the improved annotation of their area of expertise should contact the authors, or members of the GO Consortium, to discuss the options available.

## Materials and Methods

### Determination of number of annotations per protein

The QuickGO tool at http://www.ebi.ac.uk/QuickGO [5], which supplies a comprehensive set of GO annotations for UniProtKB proteins, was used to report the number of manual annotations per proteins on 11^th^ June 2011. This was achieved by filtering for all manual human protein annotations in the UniProt GOA dataset (i.e. restricted to taxon ID 9606 and all manual evidence codes). The number of manual annotations per human protein in the focused Cardiovascular and Renal Initiatives prioritised protein lists was identified by including the ID filter and selecting the BHF-UCL and KRUK protein lists.

### Determination of GO term granularity

Granularity of annotations were measured by calculating the maximum distance of a GO term from the root node terms either ‘*GO:0008150 biological_process*’, ‘*GO:0003674 molecular_function*’ and ‘*GO:0005575 cellular_component*’ using the transitive ‘*is a*’ and ‘*part of*’ GO relationships. Root node terms were given a granularity score of one and direct descendant terms a score of two. Therefore, as an example, a term supplied with granularity score of eight will have seven terms between it and the root term as measured using the connecting path in the ontology. The measurement of granularity was based on the Gene Ontology CVS revision 4.1033 (July 8^th^ 2011) at http://cvsweb.geneontology.org/cgi-bin/cvsweb.cgi/go/ontology/gene_ontology.obo and the annotation file gene_association.goa_human.99.gz (June 25^th^ 2011).

### Statistical analysis

The differing granularity distributions of GO term annotations created by the Cardiovascular and Renal Initiatives and by the other human protein annotation efforts which are not system focused were compared using the Mann Whitney U test.

### Microarray datasets used for analysis

The PAH-SSc dataset analysed used the 271 gene IDs identified by microarray analysis of peripheral blood mononuclear cells from 5 normal versus 10 PAH-SSc patients using Affymetrix GeneChip HG_U133A_2.0 The PAH-SSc-associated genes were identified by filtering for a 2.45 fold-change and 1% false discovery rate [22].

The macrophage microarray dataset analysed used the 342 gene IDs identified as differentially expressed in peritoneal resolving macrophages (n = 6) versus peritoneal naïve (n = 6) and pro-inflammatory (n = 6) macrophages using Affymetrix GeneChip Mouse Genome 430 2.0 Array. Data is located in GeneXpress, accession number E-MEXP-3189. The resolving macrophage differentially expressed genes were identified by filtering for 1.5 fold-change and 5% false discovery rate [26].

### GO analysis tools

The background ‘population’ set used for all analyses of the PAH-SSC dataset was the reviewed set of human proteins with the ‘Complete proteome’ keyword in UniProtKB (obtained on 1^st^ April 2011) with the exception of ProfCom GO which used all the proteins present in the annotation file being analysed. The mouse ‘population’ set used was the reviewed set of mouse proteins with the ‘Complete proteome’ keyword in UniProtKB (obtained on 6th April 2011).

Reanalysis of the overexpression of GO terms was performed using Ontologizer (http://compbio.charite.de/index.php/ontologizer2.html [24]), ProfCom GO (http://www.bioprofiling.de [25], [36]), and GenMAPP GO-Elite (http://www.genmapp.org/go_elite/ [23]).

GO term enrichment in Ontologizer was calculated using the parent-child intersection analysis method [37] and uses a modified Fisher's exact analysis. The single-step minP procedure of Westfall-Young was applied as multiple testing correction. Terms were considered significantly enriched if the adjusted p-value was <0.1.

BioProfiling.de (http://bioprofiling.de/) [25] provides an analytical toolkit for the interpretation of a gene/protein list. The gene list is profiled with respect to the most information available regarding gene function, protein interactions, pathway relationships, in silico predicted microRNA to gene associations, as well as information collected by text mining. This study has made use of the gene function (GO) profiling tool ProfCom [36]. Term enrichment in ProfCom GO was performed on annotation files described in the ‘Data files’ section below. ProfCom GO uses the Monte-Carlo simulation approach for multiple testing correction and hypergeometric/greedy heuristics. Terms were considered significantly enriched if the p-value was <0.01.

For GO-Elite analysis (http://www.genmapp.org/go_elite/ [23]) we used the EnsMart62plus database version with a z-score cut-off of >1.96, the minimum number of changed genes was set at 3 and the permuted p-value cut-off was <0.05. GO-Elite uses the Z-score/hypergeometric statistical method and Benjamini-Hochberg correction for multiple testing correction.

### Data files

Ontology files were downloaded from: http://cvsweb.geneontology.org/cgi-bin/cvsweb.cgi/go/ontology/gene_ontology.obo Gene Association Files (GO annotation datasets) were downloaded from: ftp://ftp.ebi.ac.uk/pub/databases/GO/goa/old/HUMAN/ or ftp://ftp.ebi.ac.uk/pub/databases/GO/goa/old/MOUSE/.

#### Files used for the analysis reported in Tables 1, 2, 3 and S1

Gene Ontology revision 4.961 (March 8^th^ 2011) and annotation file: gene_association.goa_human.95.gz (March 7^th^ 2011). For the filtered dataset the Cardiovascular Initiative (BHF-UCL) annotations were removed from this file.

#### Files used for the analysis reported in Table 4

Gene Ontology: revision 4.985 (April 13^th^ 2011) and annotation files: gene_association.goa_mouse.82.gz (April 6^th^ 2011) and annotation set; gene_association.goa_mouse.78.gz (December 13^th^ 2010).

## Supporting Information

Table S1 **Biological process GO-Elite MAPPFinder Results**.
(DOC)
