# Supplementary material for: The Impact of Focused Gene Ontology Curation of Specific Mammalian Systems
Source: PLoS One. 2011 Dec 9;6(12):e27541. doi: 10.1371/journal.pone.0027541 (PMC3235096; doi:10.1371/journal.pone.0027541)
Supplement: Table S1 — Biological process GO-Elite MAPPFinder Results. (DOC) [file pone.0027541.s001.doc]

| **GO ID** | **GO Name** | **Number Changed** | **Number Measured** | **Number in GO** | **Percent Changed** | **Percent Present** | **Z Score** | **PermuteP** | **AdjustedP** |
| --- | --- | --- | --- | --- | --- | --- | --- | --- | --- |
| GO:0002376 | immune system process | 56 | 945 | 991 | 5.93 | 95.36 | 11.3443 | 0.0000 | 0.0200 |
| GO:0006955 | immune response | 37 | 581 | 620 | 6.37 | 93.71 | 9.6816 | 0.0000 | 0.0200 |
| GO:0002684 | positive regulation of immune system process | 29 | 414 | 440 | 7.00 | 94.09 | 9.2008 | 0.0000 | 0.0200 |
| GO:0045429 | positive regulation of nitric oxide biosynthetic process | 6 | 25 | 26 | 24.00 | 96.15 | 9.1698 | 0.0000 | 0.0200 |
| GO:0002682 | regulation of immune system process | 38 | 657 | 684 | 5.78 | 96.05 | 9.0733 | 0.0000 | 0.0200 |
| GO:0051258 | protein polymerization | 8 | 44 | 46 | 18.18 | 95.65 | 9.0206 | 0.0000 | 0.0200 |
| GO:0034097 | response to cytokine stimulus | 23 | 292 | 293 | 7.88 | 99.66 | 8.9245 | 0.0000 | 0.0200 |
| GO:0045080 | positive regulation of chemokine biosynthetic process | 3 | 7 | 7 | 42.86 | 100.00 | 8.9190 | 0.0000 | 0.0200 |
| GO:0051412 | response to corticosterone stimulus | 5 | 19 | 19 | 26.32 | 100.00 | 8.8163 | 0.0000 | 0.0200 |
| GO:0048661 | positive regulation of smooth muscle cell proliferation | 6 | 27 | 27 | 22.22 | 100.00 | 8.7762 | 0.0000 | 0.0200 |
| GO:0045768 | positive regulation of anti-apoptosis | 7 | 36 | 36 | 19.44 | 100.00 | 8.7760 | 0.0000 | 0.0200 |
| GO:0002237 | response to molecule of bacterial origin | 15 | 147 | 149 | 10.20 | 98.66 | 8.6161 | 0.0000 | 0.0200 |
| GO:0050896 | response to stimulus | 146 | 5410 | 5551 | 2.70 | 97.46 | 8.4935 | 0.0000 | 0.0200 |
| GO:0048660 | regulation of smooth muscle cell proliferation | 8 | 49 | 49 | 16.33 | 100.00 | 8.4602 | 0.0000 | 0.0200 |
| GO:0042221 | response to chemical stimulus | 74 | 1995 | 2012 | 3.71 | 99.16 | 8.4554 | 0.0000 | 0.0200 |
| GO:0032496 | response to lipopolysaccharide | 14 | 134 | 136 | 10.45 | 98.53 | 8.4541 | 0.0000 | 0.0200 |
| GO:0042345 | regulation of NF-kappaB import into nucleus | 6 | 29 | 29 | 20.69 | 100.00 | 8.4224 | 0.0000 | 0.0200 |
| GO:0010033 | response to organic substance | 52 | 1172 | 1184 | 4.44 | 98.99 | 8.4161 | 0.0000 | 0.0200 |
| GO:0050776 | regulation of immune response | 27 | 421 | 448 | 6.41 | 93.97 | 8.2781 | 0.0000 | 0.0200 |
| GO:0050995 | negative regulation of lipid catabolic process | 4 | 14 | 14 | 28.57 | 100.00 | 8.2553 | 0.0000 | 0.0200 |
| GO:0007166 | cell surface receptor linked signaling pathway | 64 | 1648 | 1660 | 3.88 | 99.28 | 8.2052 | 0.0000 | 0.0200 |
| GO:0065007 | biological regulation | 175 | 7271 | 7419 | 2.41 | 98.01 | 8.0596 | 0.0000 | 0.0200 |
| GO:0009605 | response to external stimulus | 42 | 885 | 888 | 4.75 | 99.66 | 8.0181 | 0.0000 | 0.0200 |
| GO:0045767 | regulation of anti-apoptosis | 7 | 44 | 44 | 15.91 | 100.00 | 7.7896 | 0.0000 | 0.0200 |
| GO:0010470 | regulation of gastrulation | 3 | 9 | 9 | 33.33 | 100.00 | 7.7832 | 0.0000 | 0.0200 |
| GO:0046902 | regulation of mitochondrial membrane permeability | 3 | 9 | 9 | 33.33 | 100.00 | 7.7832 | 0.0000 | 0.0200 |
| GO:0031620 | regulation of fever generation | 3 | 9 | 9 | 33.33 | 100.00 | 7.7832 | 0.0005 | 0.0200 |
| GO:0031622 | positive regulation of fever generation | 3 | 9 | 9 | 33.33 | 100.00 | 7.7832 | 0.0005 | 0.0200 |
| GO:0048518 | positive regulation of biological process | 81 | 2451 | 2487 | 3.30 | 98.55 | 7.7524 | 0.0000 | 0.0200 |
| GO:0051384 | response to glucocorticoid stimulus | 12 | 117 | 117 | 10.26 | 100.00 | 7.7264 | 0.0000 | 0.0200 |
| GO:0045428 | regulation of nitric oxide biosynthetic process | 6 | 34 | 35 | 17.65 | 97.14 | 7.6727 | 0.0000 | 0.0200 |
| GO:0006950 | response to stress | 64 | 1751 | 1797 | 3.66 | 97.44 | 7.6633 | 0.0000 | 0.0200 |
| GO:0051716 | cellular response to stimulus | 105 | 3622 | 3658 | 2.90 | 99.02 | 7.5808 | 0.0000 | 0.0200 |
| GO:0051385 | response to mineralocorticoid stimulus | 5 | 25 | 25 | 20.00 | 100.00 | 7.5376 | 0.0000 | 0.0200 |
| GO:0048522 | positive regulation of cellular process | 74 | 2198 | 2215 | 3.37 | 99.23 | 7.5326 | 0.0000 | 0.0200 |
| GO:0006928 | cellular component movement | 28 | 503 | 506 | 5.57 | 99.41 | 7.4989 | 0.0000 | 0.0200 |
| GO:0050794 | regulation of cellular process | 157 | 6465 | 6583 | 2.43 | 98.21 | 7.4874 | 0.0000 | 0.0200 |
| GO:0042110 | T cell activation | 11 | 106 | 107 | 10.38 | 99.07 | 7.4537 | 0.0000 | 0.0200 |
| GO:0050789 | regulation of biological process | 163 | 6843 | 6986 | 2.38 | 97.95 | 7.4388 | 0.0000 | 0.0200 |
| GO:0009611 | response to wounding | 23 | 371 | 376 | 6.20 | 98.67 | 7.4198 | 0.0000 | 0.0200 |
| GO:0000060 | protein import into nucleus, translocation | 4 | 17 | 17 | 23.53 | 100.00 | 7.4015 | 0.0000 | 0.0200 |
| GO:0002675 | positive regulation of acute inflammatory response | 4 | 17 | 18 | 23.53 | 94.44 | 7.4015 | 0.0000 | 0.0200 |
| GO:0007165 | signal transduction | 92 | 3061 | 3089 | 3.01 | 99.09 | 7.3596 | 0.0000 | 0.0200 |
| GO:0051239 | regulation of multicellular organismal process | 48 | 1183 | 1191 | 4.06 | 99.33 | 7.3557 | 0.0000 | 0.0200 |
| GO:0031960 | response to corticosteroid stimulus | 12 | 126 | 126 | 9.52 | 100.00 | 7.3471 | 0.0000 | 0.0200 |
| GO:0001919 | regulation of receptor recycling | 3 | 10 | 10 | 30.00 | 100.00 | 7.3447 | 0.0000 | 0.0200 |
| GO:0010543 | regulation of platelet activation | 3 | 10 | 10 | 30.00 | 100.00 | 7.3447 | 0.0005 | 0.0200 |
| GO:0045073 | regulation of chemokine biosynthetic process | 3 | 10 | 10 | 30.00 | 100.00 | 7.3447 | 0.0010 | 0.0344 |
| GO:0050867 | positive regulation of cell activation | 15 | 185 | 191 | 8.11 | 96.86 | 7.3392 | 0.0000 | 0.0200 |
| GO:0032729 | positive regulation of interferon-gamma production | 5 | 27 | 27 | 18.52 | 100.00 | 7.2056 | 0.0000 | 0.0200 |
| GO:0048519 | negative regulation of biological process | 70 | 2104 | 2121 | 3.33 | 99.20 | 7.1881 | 0.0000 | 0.0200 |
| GO:0045069 | regulation of viral genome replication | 4 | 18 | 18 | 22.22 | 100.00 | 7.1638 | 0.0000 | 0.0200 |
| GO:0006952 | defense response | 32 | 656 | 684 | 4.88 | 95.91 | 7.1369 | 0.0000 | 0.0200 |
| GO:0019369 | arachidonic acid metabolic process | 3 | 11 | 11 | 27.27 | 100.00 | 6.9655 | 0.0000 | 0.0200 |
| GO:0031650 | regulation of heat generation | 3 | 11 | 11 | 27.27 | 100.00 | 6.9655 | 0.0010 | 0.0344 |
| GO:0031652 | positive regulation of heat generation | 3 | 11 | 11 | 27.27 | 100.00 | 6.9655 | 0.0010 | 0.0344 |
| GO:0042346 | positive regulation of NF-kappaB import into nucleus | 4 | 19 | 19 | 21.05 | 100.00 | 6.9444 | 0.0005 | 0.0200 |
| GO:0050778 | positive regulation of immune response | 18 | 271 | 297 | 6.64 | 91.25 | 6.9216 | 0.0000 | 0.0200 |
| GO:0065008 | regulation of biological quality | 60 | 1743 | 1759 | 3.44 | 99.09 | 6.8797 | 0.0000 | 0.0200 |
| GO:0031325 | positive regulation of cellular metabolic process | 41 | 996 | 1004 | 4.12 | 99.20 | 6.8666 | 0.0000 | 0.0200 |
| GO:0050865 | regulation of cell activation | 17 | 250 | 256 | 6.80 | 97.66 | 6.8490 | 0.0000 | 0.0200 |
| GO:0002696 | positive regulation of leukocyte activation | 14 | 181 | 187 | 7.73 | 96.79 | 6.8469 | 0.0000 | 0.0200 |
| GO:0051051 | negative regulation of transport | 14 | 181 | 182 | 7.73 | 99.45 | 6.8469 | 0.0000 | 0.0200 |
| GO:0040011 | locomotion | 35 | 788 | 791 | 4.44 | 99.62 | 6.8308 | 0.0000 | 0.0200 |
| GO:0042108 | positive regulation of cytokine biosynthetic process | 7 | 55 | 55 | 12.73 | 100.00 | 6.7845 | 0.0000 | 0.0200 |
| GO:0002673 | regulation of acute inflammatory response | 5 | 30 | 31 | 16.67 | 96.77 | 6.7681 | 0.0005 | 0.0200 |
| GO:0031323 | regulation of cellular metabolic process | 91 | 3197 | 3280 | 2.85 | 97.47 | 6.7444 | 0.0000 | 0.0200 |
| GO:0045907 | positive regulation of vasoconstriction | 4 | 20 | 20 | 20.00 | 100.00 | 6.7409 | 0.0000 | 0.0200 |
| GO:0009607 | response to biotic stimulus | 25 | 477 | 485 | 5.24 | 98.35 | 6.7088 | 0.0000 | 0.0200 |
| GO:0051591 | response to cAMP | 7 | 56 | 56 | 12.50 | 100.00 | 6.7072 | 0.0000 | 0.0200 |
| GO:0071345 | cellular response to cytokine stimulus | 15 | 209 | 210 | 7.18 | 99.52 | 6.7019 | 0.0000 | 0.0200 |
| GO:0048523 | negative regulation of cellular process | 63 | 1916 | 1930 | 3.29 | 99.27 | 6.6697 | 0.0000 | 0.0200 |
| GO:0051953 | negative regulation of amine transport | 3 | 12 | 12 | 25.00 | 100.00 | 6.6332 | 0.0005 | 0.0200 |
| GO:0032673 | regulation of interleukin-4 production | 3 | 12 | 12 | 25.00 | 100.00 | 6.6332 | 0.0010 | 0.0344 |
| GO:0032715 | negative regulation of interleukin-6 production | 3 | 12 | 12 | 25.00 | 100.00 | 6.6332 | 0.0010 | 0.0344 |
| GO:0019221 | cytokine-mediated signaling pathway | 14 | 189 | 190 | 7.41 | 99.47 | 6.6291 | 0.0000 | 0.0200 |
| GO:0030335 | positive regulation of cell migration | 12 | 146 | 147 | 8.22 | 99.32 | 6.6223 | 0.0000 | 0.0200 |
| GO:2000147 | positive regulation of cell motility | 12 | 146 | 147 | 8.22 | 99.32 | 6.6223 | 0.0000 | 0.0200 |
| GO:0009987 | cellular process | 215 | 10711 | 10882 | 2.01 | 98.43 | 6.6129 | 0.0000 | 0.0200 |
| GO:0001775 | cell activation | 24 | 455 | 460 | 5.27 | 98.91 | 6.6073 | 0.0000 | 0.0200 |
| GO:0006954 | inflammatory response | 17 | 262 | 265 | 6.49 | 98.87 | 6.5997 | 0.0000 | 0.0200 |
| GO:0019222 | regulation of metabolic process | 97 | 3544 | 3631 | 2.74 | 97.60 | 6.5947 | 0.0000 | 0.0200 |
| GO:0009893 | positive regulation of metabolic process | 42 | 1073 | 1081 | 3.91 | 99.26 | 6.5862 | 0.0000 | 0.0200 |
| GO:0051251 | positive regulation of lymphocyte activation | 13 | 169 | 175 | 7.69 | 96.57 | 6.5684 | 0.0000 | 0.0200 |
| GO:0080090 | regulation of primary metabolic process | 89 | 3165 | 3245 | 2.81 | 97.53 | 6.5281 | 0.0000 | 0.0200 |
| GO:0016477 | cell migration | 21 | 377 | 378 | 5.57 | 99.74 | 6.4736 | 0.0000 | 0.0200 |
| GO:0043623 | cellular protein complex assembly | 12 | 151 | 154 | 7.95 | 98.05 | 6.4618 | 0.0000 | 0.0200 |
| GO:0050900 | leukocyte migration | 12 | 151 | 152 | 7.95 | 99.34 | 6.4618 | 0.0000 | 0.0200 |
| GO:0060255 | regulation of macromolecule metabolic process | 85 | 2989 | 3064 | 2.84 | 97.55 | 6.4603 | 0.0000 | 0.0200 |
| GO:0040017 | positive regulation of locomotion | 12 | 152 | 153 | 7.89 | 99.35 | 6.4306 | 0.0000 | 0.0200 |
| GO:0002819 | regulation of adaptive immune response | 7 | 60 | 61 | 11.67 | 98.36 | 6.4161 | 0.0000 | 0.0200 |
| GO:0045321 | leukocyte activation | 15 | 222 | 224 | 6.76 | 99.11 | 6.3959 | 0.0000 | 0.0200 |
| GO:0050684 | regulation of mRNA processing | 4 | 22 | 22 | 18.18 | 100.00 | 6.3744 | 0.0000 | 0.0200 |
| GO:0043392 | negative regulation of DNA binding | 3 | 13 | 13 | 23.08 | 100.00 | 6.3386 | 0.0005 | 0.0200 |
| GO:0010575 | positive regulation vascular endothelial growth factor production | 3 | 13 | 13 | 23.08 | 100.00 | 6.3386 | 0.0015 | 0.0482 |
| GO:0048583 | regulation of response to stimulus | 55 | 1641 | 1678 | 3.35 | 97.79 | 6.3383 | 0.0000 | 0.0200 |
| GO:0042327 | positive regulation of phosphorylation | 13 | 178 | 180 | 7.30 | 98.89 | 6.3175 | 0.0000 | 0.0200 |
| GO:0046649 | lymphocyte activation | 13 | 178 | 180 | 7.30 | 98.89 | 6.3175 | 0.0000 | 0.0200 |
| GO:0051272 | positive regulation of cellular component movement | 12 | 156 | 157 | 7.69 | 99.36 | 6.3083 | 0.0000 | 0.0200 |
| GO:0001525 | angiogenesis | 11 | 135 | 137 | 8.15 | 98.54 | 6.2984 | 0.0000 | 0.0200 |
| GO:0042990 | regulation of transcription factor import into nucleus | 6 | 47 | 47 | 12.77 | 100.00 | 6.2919 | 0.0000 | 0.0200 |
| GO:0061041 | regulation of wound healing | 6 | 47 | 49 | 12.77 | 95.92 | 6.2919 | 0.0000 | 0.0200 |
| GO:0008284 | positive regulation of cell proliferation | 24 | 483 | 489 | 4.97 | 98.77 | 6.2574 | 0.0000 | 0.0200 |
| GO:0010562 | positive regulation of phosphorus metabolic process | 13 | 182 | 184 | 7.14 | 98.91 | 6.2113 | 0.0000 | 0.0200 |
| GO:0045937 | positive regulation of phosphate metabolic process | 13 | 182 | 184 | 7.14 | 98.91 | 6.2113 | 0.0000 | 0.0200 |
| GO:0042060 | wound healing | 6 | 48 | 50 | 12.50 | 96.00 | 6.2082 | 0.0000 | 0.0200 |
| GO:0001934 | positive regulation of protein phosphorylation | 12 | 162 | 164 | 7.41 | 98.78 | 6.1325 | 0.0000 | 0.0200 |
| GO:0070887 | cellular response to chemical stimulus | 31 | 729 | 735 | 4.25 | 99.18 | 6.1325 | 0.0000 | 0.0200 |
| GO:0042981 | regulation of apoptosis | 37 | 949 | 954 | 3.90 | 99.48 | 6.1304 | 0.0000 | 0.0200 |
| GO:0032502 | developmental process | 87 | 3192 | 3216 | 2.73 | 99.25 | 6.1208 | 0.0000 | 0.0200 |
| GO:0001817 | regulation of cytokine production | 16 | 262 | 264 | 6.11 | 99.24 | 6.0920 | 0.0000 | 0.0200 |
| GO:0048545 | response to steroid hormone stimulus | 16 | 262 | 264 | 6.11 | 99.24 | 6.0920 | 0.0000 | 0.0200 |
| GO:0010888 | negative regulation of lipid storage | 3 | 14 | 14 | 21.43 | 100.00 | 6.0749 | 0.0000 | 0.0200 |
| GO:0002286 | T cell activation involved in immune response | 3 | 14 | 14 | 21.43 | 100.00 | 6.0749 | 0.0005 | 0.0200 |
| GO:0042116 | macrophage activation | 3 | 14 | 14 | 21.43 | 100.00 | 6.0749 | 0.0010 | 0.0344 |
| GO:0030728 | ovulation | 3 | 14 | 14 | 21.43 | 100.00 | 6.0749 | 0.0030 | 0.0813 |
| GO:0043067 | regulation of programmed cell death | 37 | 959 | 964 | 3.86 | 99.48 | 6.0588 | 0.0000 | 0.0200 |
| GO:0001816 | cytokine production | 4 | 24 | 24 | 16.67 | 100.00 | 6.0525 | 0.0005 | 0.0200 |
| GO:0002694 | regulation of leukocyte activation | 15 | 239 | 245 | 6.28 | 97.55 | 6.0294 | 0.0000 | 0.0200 |
| GO:0051240 | positive regulation of multicellular organismal process | 18 | 321 | 323 | 5.61 | 99.38 | 6.0184 | 0.0000 | 0.0200 |
| GO:0043065 | positive regulation of apoptosis | 23 | 473 | 475 | 4.86 | 99.58 | 5.9991 | 0.0000 | 0.0200 |
| GO:0048870 | cell motility | 21 | 413 | 416 | 5.08 | 99.28 | 5.9683 | 0.0000 | 0.0200 |
| GO:0010941 | regulation of cell death | 37 | 974 | 979 | 3.80 | 99.49 | 5.9532 | 0.0000 | 0.0200 |
| GO:0022407 | regulation of cell-cell adhesion | 5 | 37 | 38 | 13.51 | 97.37 | 5.9522 | 0.0000 | 0.0200 |
| GO:0043068 | positive regulation of programmed cell death | 23 | 477 | 479 | 4.82 | 99.58 | 5.9515 | 0.0000 | 0.0200 |
| GO:0050793 | regulation of developmental process | 35 | 899 | 906 | 3.89 | 99.23 | 5.9434 | 0.0000 | 0.0200 |
| GO:0010604 | positive regulation of macromolecule metabolic process | 37 | 976 | 982 | 3.79 | 99.39 | 5.9392 | 0.0000 | 0.0200 |
| GO:0051249 | regulation of lymphocyte activation | 14 | 218 | 224 | 6.42 | 97.32 | 5.9316 | 0.0000 | 0.0200 |
| GO:0009991 | response to extracellular stimulus | 16 | 271 | 273 | 5.90 | 99.27 | 5.9229 | 0.0000 | 0.0200 |
| GO:0002757 | immune response-activating signal transduction | 12 | 172 | 178 | 6.98 | 96.63 | 5.8579 | 0.0000 | 0.0200 |
| GO:0010942 | positive regulation of cell death | 23 | 485 | 487 | 4.74 | 99.59 | 5.8577 | 0.0000 | 0.0200 |
| GO:0010574 | regulation of vascular endothelial growth factor production | 3 | 15 | 15 | 20.00 | 100.00 | 5.8369 | 0.0025 | 0.0712 |
| GO:0014070 | response to organic cyclic compound | 12 | 174 | 177 | 6.90 | 98.31 | 5.8055 | 0.0000 | 0.0200 |
| GO:0045637 | regulation of myeloid cell differentiation | 8 | 88 | 88 | 9.09 | 100.00 | 5.8012 | 0.0005 | 0.0200 |
| GO:0032570 | response to progesterone stimulus | 4 | 26 | 26 | 15.38 | 100.00 | 5.7666 | 0.0000 | 0.0200 |
| GO:0042993 | positive regulation of transcription factor import into nucleus | 4 | 26 | 26 | 15.38 | 100.00 | 5.7666 | 0.0005 | 0.0200 |
| GO:0001819 | positive regulation of cytokine production | 10 | 130 | 132 | 7.69 | 98.48 | 5.7543 | 0.0000 | 0.0200 |
| GO:0002764 | immune response-regulating signaling pathway | 12 | 176 | 182 | 6.82 | 96.70 | 5.7539 | 0.0000 | 0.0200 |
| GO:0001818 | negative regulation of cytokine production | 8 | 90 | 90 | 8.89 | 100.00 | 5.7104 | 0.0000 | 0.0200 |
| GO:0042127 | regulation of cell proliferation | 34 | 894 | 902 | 3.80 | 99.11 | 5.7003 | 0.0000 | 0.0200 |
| GO:0019220 | regulation of phosphate metabolic process | 27 | 636 | 643 | 4.25 | 98.91 | 5.6969 | 0.0000 | 0.0200 |
| GO:0051174 | regulation of phosphorus metabolic process | 27 | 636 | 643 | 4.25 | 98.91 | 5.6969 | 0.0000 | 0.0200 |
| GO:0071496 | cellular response to external stimulus | 10 | 132 | 133 | 7.58 | 99.25 | 5.6891 | 0.0000 | 0.0200 |
| GO:0048872 | homeostasis of number of cells | 5 | 40 | 41 | 12.50 | 97.56 | 5.6660 | 0.0005 | 0.0200 |
| GO:0000038 | very long-chain fatty acid metabolic process | 4 | 27 | 27 | 14.81 | 100.00 | 5.6349 | 0.0005 | 0.0200 |
| GO:0002449 | lymphocyte mediated immunity | 4 | 27 | 28 | 14.81 | 96.43 | 5.6349 | 0.0005 | 0.0200 |
| GO:0006692 | prostanoid metabolic process | 4 | 27 | 27 | 14.81 | 100.00 | 5.6349 | 0.0010 | 0.0344 |
| GO:0051241 | negative regulation of multicellular organismal process | 14 | 233 | 236 | 6.01 | 98.73 | 5.6168 | 0.0000 | 0.0200 |
| GO:0001932 | regulation of protein phosphorylation | 25 | 578 | 583 | 4.33 | 99.14 | 5.5809 | 0.0000 | 0.0200 |
| GO:0019229 | regulation of vasoconstriction | 5 | 41 | 41 | 12.20 | 100.00 | 5.5771 | 0.0000 | 0.0200 |
| GO:0051246 | regulation of protein metabolic process | 37 | 1030 | 1038 | 3.59 | 99.23 | 5.5750 | 0.0000 | 0.0200 |
| GO:0031349 | positive regulation of defense response | 11 | 159 | 160 | 6.92 | 99.38 | 5.5696 | 0.0000 | 0.0200 |
| GO:0031399 | regulation of protein modification process | 29 | 726 | 731 | 3.99 | 99.32 | 5.5402 | 0.0000 | 0.0200 |
| GO:0051094 | positive regulation of developmental process | 20 | 416 | 418 | 4.81 | 99.52 | 5.5240 | 0.0000 | 0.0200 |
| GO:0002253 | activation of immune response | 13 | 212 | 237 | 6.13 | 89.45 | 5.5020 | 0.0000 | 0.0200 |
| GO:0032844 | regulation of homeostatic process | 10 | 138 | 140 | 7.25 | 98.57 | 5.5012 | 0.0000 | 0.0200 |
| GO:0050871 | positive regulation of B cell activation | 5 | 42 | 43 | 11.90 | 97.67 | 5.4913 | 0.0000 | 0.0200 |
| GO:0050671 | positive regulation of lymphocyte proliferation | 7 | 76 | 77 | 9.21 | 98.70 | 5.4745 | 0.0000 | 0.0200 |
| GO:2000026 | regulation of multicellular organismal development | 28 | 698 | 703 | 4.01 | 99.29 | 5.4650 | 0.0000 | 0.0200 |
| GO:0071310 | cellular response to organic substance | 25 | 590 | 594 | 4.24 | 99.33 | 5.4633 | 0.0000 | 0.0200 |
| GO:0010243 | response to organic nitrogen | 8 | 96 | 96 | 8.33 | 100.00 | 5.4536 | 0.0000 | 0.0200 |
| GO:0032103 | positive regulation of response to external stimulus | 8 | 96 | 97 | 8.33 | 98.97 | 5.4536 | 0.0000 | 0.0200 |
| GO:0032268 | regulation of cellular protein metabolic process | 34 | 929 | 936 | 3.66 | 99.25 | 5.4509 | 0.0000 | 0.0200 |
| GO:0044092 | negative regulation of molecular function | 22 | 488 | 492 | 4.51 | 99.19 | 5.4485 | 0.0000 | 0.0200 |
| GO:0032946 | positive regulation of mononuclear cell proliferation | 7 | 77 | 78 | 9.09 | 98.72 | 5.4248 | 0.0000 | 0.0200 |
| GO:0002822 | regulation of adaptive immune response based on somatic recombination of immune receptors built from immunoglobulin superfamily domains | 6 | 59 | 60 | 10.17 | 98.33 | 5.4228 | 0.0000 | 0.0200 |
| GO:0010039 | response to iron ion | 3 | 17 | 17 | 17.65 | 100.00 | 5.4227 | 0.0005 | 0.0200 |
| GO:0033628 | regulation of cell adhesion mediated by integrin | 3 | 17 | 17 | 17.65 | 100.00 | 5.4227 | 0.0020 | 0.0604 |
| GO:0050766 | positive regulation of phagocytosis | 3 | 17 | 17 | 17.65 | 100.00 | 5.4227 | 0.0020 | 0.0604 |
| GO:0030155 | regulation of cell adhesion | 11 | 165 | 167 | 6.67 | 98.80 | 5.4099 | 0.0000 | 0.0200 |
| GO:0030334 | regulation of cell migration | 14 | 244 | 246 | 5.74 | 99.19 | 5.4022 | 0.0000 | 0.0200 |
| GO:0007044 | cell-substrate junction assembly | 4 | 29 | 29 | 13.79 | 100.00 | 5.3912 | 0.0010 | 0.0344 |
| GO:0043271 | negative regulation of ion transport | 4 | 29 | 29 | 13.79 | 100.00 | 5.3912 | 0.0010 | 0.0344 |
| GO:0030890 | positive regulation of B cell proliferation | 4 | 29 | 30 | 13.79 | 96.67 | 5.3912 | 0.0015 | 0.0482 |
| GO:0045595 | regulation of cell differentiation | 26 | 635 | 639 | 4.09 | 99.37 | 5.3765 | 0.0000 | 0.0200 |
| GO:0070665 | positive regulation of leukocyte proliferation | 7 | 78 | 79 | 8.97 | 98.73 | 5.3759 | 0.0000 | 0.0200 |
| GO:2000145 | regulation of cell motility | 14 | 247 | 249 | 5.67 | 99.20 | 5.3459 | 0.0000 | 0.0200 |
| GO:0009891 | positive regulation of biosynthetic process | 27 | 681 | 685 | 3.96 | 99.42 | 5.2939 | 0.0000 | 0.0200 |
| GO:0042035 | regulation of cytokine biosynthetic process | 7 | 80 | 80 | 8.75 | 100.00 | 5.2807 | 0.0000 | 0.0200 |
| GO:0046330 | positive regulation of JNK cascade | 4 | 30 | 30 | 13.33 | 100.00 | 5.2780 | 0.0005 | 0.0200 |
| GO:0050994 | regulation of lipid catabolic process | 4 | 30 | 31 | 13.33 | 96.77 | 5.2780 | 0.0015 | 0.0482 |
| GO:0032642 | regulation of chemokine production | 4 | 30 | 30 | 13.33 | 100.00 | 5.2780 | 0.0020 | 0.0604 |
| GO:0034622 | cellular macromolecular complex assembly | 17 | 340 | 348 | 5.00 | 97.70 | 5.2748 | 0.0000 | 0.0200 |
| GO:0032675 | regulation of interleukin-6 production | 5 | 45 | 46 | 11.11 | 97.83 | 5.2498 | 0.0010 | 0.0344 |
| GO:0010876 | lipid localization | 3 | 18 | 18 | 16.67 | 100.00 | 5.2407 | 0.0015 | 0.0482 |
| GO:0019915 | lipid storage | 3 | 18 | 18 | 16.67 | 100.00 | 5.2407 | 0.0015 | 0.0482 |
| GO:0022409 | positive regulation of cell-cell adhesion | 3 | 18 | 18 | 16.67 | 100.00 | 5.2407 | 0.0025 | 0.0712 |
| GO:0032101 | regulation of response to external stimulus | 13 | 225 | 228 | 5.78 | 98.68 | 5.2341 | 0.0000 | 0.0200 |
| GO:0042325 | regulation of phosphorylation | 25 | 616 | 621 | 4.06 | 99.19 | 5.2181 | 0.0000 | 0.0200 |
| GO:0009612 | response to mechanical stimulus | 8 | 102 | 103 | 7.84 | 99.03 | 5.2175 | 0.0005 | 0.0200 |
| GO:0051084 | 'de novo' posttranslational protein folding | 5 | 46 | 46 | 10.87 | 100.00 | 5.1742 | 0.0010 | 0.0344 |
| GO:0002460 | adaptive immune response based on somatic recombination of immune receptors built from immunoglobulin superfamily domains | 4 | 31 | 32 | 12.90 | 96.88 | 5.1699 | 0.0000 | 0.0200 |
| GO:0031328 | positive regulation of cellular biosynthetic process | 26 | 661 | 665 | 3.93 | 99.40 | 5.1454 | 0.0000 | 0.0200 |
| GO:0007596 | blood coagulation | 20 | 448 | 452 | 4.46 | 99.12 | 5.1374 | 0.0000 | 0.0200 |
| GO:0050817 | coagulation | 20 | 448 | 452 | 4.46 | 99.12 | 5.1374 | 0.0000 | 0.0200 |
| GO:0051247 | positive regulation of protein metabolic process | 19 | 415 | 418 | 4.58 | 99.28 | 5.1315 | 0.0000 | 0.0200 |
| GO:0007599 | hemostasis | 20 | 451 | 455 | 4.43 | 99.12 | 5.1029 | 0.0000 | 0.0200 |
| GO:0032649 | regulation of interferon-gamma production | 5 | 47 | 47 | 10.64 | 100.00 | 5.1008 | 0.0005 | 0.0200 |
| GO:0050729 | positive regulation of inflammatory response | 5 | 47 | 48 | 10.64 | 97.92 | 5.1008 | 0.0005 | 0.0200 |
| GO:0030098 | lymphocyte differentiation | 7 | 84 | 84 | 8.33 | 100.00 | 5.0995 | 0.0000 | 0.0200 |
| GO:0034341 | response to interferon-gamma | 7 | 84 | 85 | 8.33 | 98.82 | 5.0995 | 0.0005 | 0.0200 |
| GO:0006461 | protein complex assembly | 20 | 452 | 457 | 4.42 | 98.91 | 5.0915 | 0.0000 | 0.0200 |
| GO:0048584 | positive regulation of response to stimulus | 28 | 744 | 771 | 3.76 | 96.50 | 5.0861 | 0.0000 | 0.0200 |
| GO:0002756 | MyD88-independent toll-like receptor signaling pathway | 6 | 65 | 65 | 9.23 | 100.00 | 5.0745 | 0.0000 | 0.0200 |
| GO:0051702 | interaction with symbiont | 3 | 19 | 19 | 15.79 | 100.00 | 5.0725 | 0.0010 | 0.0344 |
| GO:0051926 | negative regulation of calcium ion transport | 3 | 19 | 19 | 15.79 | 100.00 | 5.0725 | 0.0025 | 0.0712 |
| GO:0001836 | release of cytochrome c from mitochondria | 3 | 19 | 19 | 15.79 | 100.00 | 5.0725 | 0.0030 | 0.0813 |
| GO:0051173 | positive regulation of nitrogen compound metabolic process | 24 | 597 | 601 | 4.02 | 99.33 | 5.0563 | 0.0000 | 0.0200 |
| GO:0009628 | response to abiotic stimulus | 20 | 457 | 459 | 4.38 | 99.56 | 5.0348 | 0.0000 | 0.0200 |
| GO:0002685 | regulation of leukocyte migration | 5 | 48 | 49 | 10.42 | 97.96 | 5.0295 | 0.0010 | 0.0344 |
| GO:0010035 | response to inorganic substance | 14 | 268 | 271 | 5.22 | 98.89 | 4.9743 | 0.0000 | 0.0200 |
| GO:0002250 | adaptive immune response | 4 | 33 | 34 | 12.12 | 97.06 | 4.9677 | 0.0000 | 0.0200 |
| GO:0070372 | regulation of ERK1 and ERK2 cascade | 5 | 49 | 49 | 10.20 | 100.00 | 4.9603 | 0.0015 | 0.0482 |
| GO:0048869 | cellular developmental process | 44 | 1440 | 1453 | 3.06 | 99.11 | 4.9421 | 0.0000 | 0.0200 |
| GO:0065003 | macromolecular complex assembly | 25 | 647 | 658 | 3.86 | 98.33 | 4.9416 | 0.0000 | 0.0200 |
| GO:0030593 | neutrophil chemotaxis | 3 | 20 | 20 | 15.00 | 100.00 | 4.9164 | 0.0010 | 0.0344 |
| GO:0010921 | regulation of phosphatase activity | 3 | 20 | 22 | 15.00 | 90.91 | 4.9164 | 0.0020 | 0.0604 |
| GO:0002260 | lymphocyte homeostasis | 3 | 20 | 21 | 15.00 | 95.24 | 4.9164 | 0.0025 | 0.0712 |
| GO:0006693 | prostaglandin metabolic process | 3 | 20 | 20 | 15.00 | 100.00 | 4.9164 | 0.0040 | 0.0989 |
| GO:0048662 | negative regulation of smooth muscle cell proliferation | 3 | 20 | 20 | 15.00 | 100.00 | 4.9164 | 0.0040 | 0.0989 |
| GO:0051270 | regulation of cellular component movement | 14 | 272 | 274 | 5.15 | 99.27 | 4.9078 | 0.0000 | 0.0200 |
| GO:0043066 | negative regulation of apoptosis | 18 | 400 | 402 | 4.50 | 99.50 | 4.9064 | 0.0000 | 0.0200 |
| GO:0045766 | positive regulation of angiogenesis | 5 | 50 | 50 | 10.00 | 100.00 | 4.8929 | 0.0005 | 0.0200 |
| GO:0040012 | regulation of locomotion | 14 | 273 | 275 | 5.13 | 99.27 | 4.8913 | 0.0000 | 0.0200 |
| GO:0002443 | leukocyte mediated immunity | 4 | 34 | 35 | 11.76 | 97.14 | 4.8729 | 0.0020 | 0.0604 |
| GO:0034130 | toll-like receptor 1 signaling pathway | 6 | 69 | 69 | 8.70 | 100.00 | 4.8657 | 0.0000 | 0.0200 |
| GO:0034134 | toll-like receptor 2 signaling pathway | 6 | 69 | 69 | 8.70 | 100.00 | 4.8657 | 0.0000 | 0.0200 |
| GO:0009889 | regulation of biosynthetic process | 63 | 2364 | 2432 | 2.66 | 97.20 | 4.8592 | 0.0000 | 0.0200 |
| GO:0043069 | negative regulation of programmed cell death | 18 | 405 | 407 | 4.44 | 99.51 | 4.8454 | 0.0000 | 0.0200 |
| GO:0006979 | response to oxidative stress | 11 | 189 | 191 | 5.82 | 98.95 | 4.8397 | 0.0000 | 0.0200 |
| GO:0048646 | anatomical structure formation involved in morphogenesis | 17 | 372 | 375 | 4.57 | 99.20 | 4.8387 | 0.0000 | 0.0200 |
| GO:0002521 | leukocyte differentiation | 8 | 113 | 113 | 7.08 | 100.00 | 4.8293 | 0.0000 | 0.0200 |
| GO:0030217 | T cell differentiation | 5 | 51 | 51 | 9.80 | 100.00 | 4.8274 | 0.0000 | 0.0200 |
| GO:0006458 | 'de novo' protein folding | 5 | 51 | 51 | 9.80 | 100.00 | 4.8274 | 0.0010 | 0.0344 |
| GO:0050870 | positive regulation of T cell activation | 9 | 138 | 143 | 6.52 | 96.50 | 4.8042 | 0.0000 | 0.0200 |
| GO:0008150 | biological_process | 243 | 13952 | 14333 | 1.74 | 97.34 | 4.7843 | 0.0140 | 0.2330 |
| GO:0051100 | negative regulation of binding | 4 | 35 | 36 | 11.43 | 97.22 | 4.7818 | 0.0025 | 0.0712 |
| GO:0070482 | response to oxygen levels | 10 | 165 | 166 | 6.06 | 99.40 | 4.7720 | 0.0000 | 0.0200 |
| GO:0033280 | response to vitamin D | 3 | 21 | 21 | 14.29 | 100.00 | 4.7708 | 0.0025 | 0.0712 |
| GO:0001776 | leukocyte homeostasis | 3 | 21 | 22 | 14.29 | 95.45 | 4.7708 | 0.0030 | 0.0813 |
| GO:0070542 | response to fatty acid | 3 | 21 | 21 | 14.29 | 100.00 | 4.7708 | 0.0040 | 0.0989 |
| GO:0031668 | cellular response to extracellular stimulus | 7 | 92 | 93 | 7.61 | 98.92 | 4.7698 | 0.0000 | 0.0200 |
| GO:0080134 | regulation of response to stress | 20 | 482 | 486 | 4.15 | 99.18 | 4.7623 | 0.0000 | 0.0200 |
| GO:0006935 | chemotaxis | 20 | 483 | 484 | 4.14 | 99.79 | 4.7518 | 0.0000 | 0.0200 |
| GO:0042330 | taxis | 20 | 483 | 484 | 4.14 | 99.79 | 4.7518 | 0.0000 | 0.0200 |
| GO:0045597 | positive regulation of cell differentiation | 15 | 314 | 315 | 4.78 | 99.68 | 4.7400 | 0.0000 | 0.0200 |
| GO:0031667 | response to nutrient levels | 13 | 253 | 254 | 5.14 | 99.61 | 4.7192 | 0.0000 | 0.0200 |
| GO:0060548 | negative regulation of cell death | 18 | 416 | 419 | 4.33 | 99.28 | 4.7145 | 0.0000 | 0.0200 |
| GO:0016265 | death | 27 | 753 | 760 | 3.59 | 99.08 | 4.7113 | 0.0000 | 0.0200 |
| GO:0044057 | regulation of system process | 15 | 317 | 318 | 4.73 | 99.69 | 4.6968 | 0.0000 | 0.0200 |
| GO:0002252 | immune effector process | 9 | 142 | 162 | 6.34 | 87.65 | 4.6946 | 0.0010 | 0.0344 |
| GO:0045638 | negative regulation of myeloid cell differentiation | 4 | 36 | 36 | 11.11 | 100.00 | 4.6943 | 0.0010 | 0.0344 |
| GO:0070304 | positive regulation of stress-activated protein kinase signaling cascade | 4 | 36 | 36 | 11.11 | 100.00 | 4.6943 | 0.0020 | 0.0604 |
| GO:0050792 | regulation of viral reproduction | 6 | 73 | 73 | 8.22 | 100.00 | 4.6727 | 0.0000 | 0.0200 |
| GO:0051092 | positive regulation of NF-kappaB transcription factor activity | 6 | 73 | 74 | 8.22 | 98.65 | 4.6727 | 0.0005 | 0.0200 |
| GO:0002755 | MyD88-dependent toll-like receptor signaling pathway | 6 | 73 | 73 | 8.22 | 100.00 | 4.6727 | 0.0010 | 0.0344 |
| GO:0007243 | intracellular protein kinase cascade | 13 | 256 | 256 | 5.08 | 100.00 | 4.6684 | 0.0000 | 0.0200 |
| GO:0050851 | antigen receptor-mediated signaling pathway | 7 | 95 | 100 | 7.37 | 95.00 | 4.6558 | 0.0005 | 0.0200 |
| GO:0043523 | regulation of neuron apoptosis | 7 | 95 | 96 | 7.37 | 98.96 | 4.6558 | 0.0010 | 0.0344 |
| GO:0032846 | positive regulation of homeostatic process | 5 | 54 | 56 | 9.26 | 96.43 | 4.6409 | 0.0005 | 0.0200 |
| GO:0050764 | regulation of phagocytosis | 3 | 22 | 22 | 13.64 | 100.00 | 4.6347 | 0.0025 | 0.0712 |
| GO:0032722 | positive regulation of chemokine production | 3 | 22 | 22 | 13.64 | 100.00 | 4.6347 | 0.0030 | 0.0813 |
| GO:0014910 | regulation of smooth muscle cell migration | 3 | 22 | 22 | 13.64 | 100.00 | 4.6347 | 0.0045 | 0.1080 |
| GO:0009314 | response to radiation | 12 | 228 | 229 | 5.26 | 99.56 | 4.6314 | 0.0000 | 0.0200 |
| GO:0006357 | regulation of transcription from RNA polymerase II promoter | 26 | 724 | 728 | 3.59 | 99.45 | 4.6280 | 0.0000 | 0.0200 |
| GO:0031401 | positive regulation of protein modification process | 14 | 290 | 292 | 4.83 | 99.32 | 4.6228 | 0.0000 | 0.0200 |
| GO:0042493 | response to drug | 13 | 259 | 265 | 5.02 | 97.74 | 4.6183 | 0.0000 | 0.0200 |
| GO:0002821 | positive regulation of adaptive immune response | 4 | 37 | 38 | 10.81 | 97.37 | 4.6101 | 0.0020 | 0.0604 |
| GO:0031326 | regulation of cellular biosynthetic process | 61 | 2335 | 2403 | 2.61 | 97.17 | 4.6018 | 0.0000 | 0.0200 |
| GO:0065009 | regulation of molecular function | 40 | 1327 | 1347 | 3.01 | 98.52 | 4.5998 | 0.0000 | 0.0200 |
| GO:0032270 | positive regulation of cellular protein metabolic process | 16 | 358 | 361 | 4.47 | 99.17 | 4.5879 | 0.0005 | 0.0200 |
| GO:0050863 | regulation of T cell activation | 10 | 173 | 178 | 5.78 | 97.19 | 4.5853 | 0.0000 | 0.0200 |
| GO:0009725 | response to hormone stimulus | 22 | 574 | 578 | 3.83 | 99.31 | 4.5822 | 0.0000 | 0.0200 |
| GO:0006916 | anti-apoptosis | 11 | 203 | 203 | 5.42 | 100.00 | 4.5487 | 0.0000 | 0.0200 |
| GO:0032879 | regulation of localization | 30 | 899 | 906 | 3.34 | 99.23 | 4.5464 | 0.0000 | 0.0200 |
| GO:0009892 | negative regulation of metabolic process | 27 | 775 | 780 | 3.48 | 99.36 | 4.5464 | 0.0000 | 0.0200 |
| GO:0008063 | Toll signaling pathway | 6 | 76 | 76 | 7.89 | 100.00 | 4.5370 | 0.0005 | 0.0200 |
| GO:0034142 | toll-like receptor 4 signaling pathway | 6 | 76 | 76 | 7.89 | 100.00 | 4.5370 | 0.0005 | 0.0200 |
| GO:0010556 | regulation of macromolecule biosynthetic process | 57 | 2154 | 2220 | 2.65 | 97.03 | 4.5299 | 0.0000 | 0.0200 |
| GO:0002761 | regulation of myeloid leukocyte differentiation | 5 | 56 | 56 | 8.93 | 100.00 | 4.5242 | 0.0005 | 0.0200 |
| GO:0051090 | regulation of sequence-specific DNA binding transcription factor activity | 12 | 234 | 237 | 5.13 | 98.73 | 4.5233 | 0.0000 | 0.0200 |
| GO:0055065 | metal ion homeostasis | 13 | 265 | 267 | 4.91 | 99.25 | 4.5202 | 0.0000 | 0.0200 |
| GO:0010557 | positive regulation of macromolecule biosynthetic process | 22 | 581 | 583 | 3.79 | 99.66 | 4.5187 | 0.0000 | 0.0200 |
| GO:0050848 | regulation of calcium-mediated signaling | 3 | 23 | 23 | 13.04 | 100.00 | 4.5070 | 0.0030 | 0.0813 |
| GO:0032755 | positive regulation of interleukin-6 production | 3 | 23 | 24 | 13.04 | 95.83 | 4.5070 | 0.0060 | 0.1345 |
| GO:0043525 | positive regulation of neuron apoptosis | 3 | 23 | 23 | 13.04 | 100.00 | 4.5070 | 0.0065 | 0.1432 |
| GO:0050878 | regulation of body fluid levels | 20 | 507 | 512 | 3.94 | 99.02 | 4.5066 | 0.0000 | 0.0200 |
| GO:0009416 | response to light stimulus | 9 | 150 | 150 | 6.00 | 100.00 | 4.4871 | 0.0000 | 0.0200 |
| GO:0008219 | cell death | 26 | 747 | 754 | 3.48 | 99.07 | 4.4522 | 0.0000 | 0.0200 |
| GO:0030193 | regulation of blood coagulation | 4 | 39 | 41 | 10.26 | 95.12 | 4.4507 | 0.0010 | 0.0344 |
| GO:0046496 | nicotinamide nucleotide metabolic process | 4 | 39 | 39 | 10.26 | 100.00 | 4.4507 | 0.0025 | 0.0712 |
| GO:0002687 | positive regulation of leukocyte migration | 4 | 39 | 40 | 10.26 | 97.50 | 4.4507 | 0.0040 | 0.0989 |
| GO:0051048 | negative regulation of secretion | 6 | 78 | 79 | 7.69 | 98.73 | 4.4505 | 0.0005 | 0.0200 |
| GO:0046822 | regulation of nucleocytoplasmic transport | 7 | 101 | 101 | 6.93 | 100.00 | 4.4417 | 0.0015 | 0.0482 |
| GO:0009719 | response to endogenous stimulus | 23 | 629 | 633 | 3.66 | 99.37 | 4.4376 | 0.0000 | 0.0200 |
| GO:0010468 | regulation of gene expression | 58 | 2235 | 2302 | 2.60 | 97.09 | 4.4153 | 0.0000 | 0.0200 |
| GO:0001666 | response to hypoxia | 9 | 153 | 154 | 5.88 | 99.35 | 4.4129 | 0.0000 | 0.0200 |
| GO:0048878 | chemical homeostasis | 20 | 519 | 525 | 3.85 | 98.86 | 4.3892 | 0.0000 | 0.0200 |
| GO:0031347 | regulation of defense response | 14 | 306 | 307 | 4.58 | 99.67 | 4.3875 | 0.0000 | 0.0200 |
| GO:0033554 | cellular response to stress | 25 | 715 | 725 | 3.50 | 98.62 | 4.3870 | 0.0000 | 0.0200 |
| GO:0045833 | negative regulation of lipid metabolic process | 4 | 40 | 40 | 10.00 | 100.00 | 4.3751 | 0.0015 | 0.0482 |
| GO:0030888 | regulation of B cell proliferation | 4 | 40 | 41 | 10.00 | 97.56 | 4.3751 | 0.0035 | 0.0911 |
| GO:0070374 | positive regulation of ERK1 and ERK2 cascade | 4 | 40 | 40 | 10.00 | 100.00 | 4.3751 | 0.0035 | 0.0911 |
| GO:0071222 | cellular response to lipopolysaccharide | 4 | 40 | 40 | 10.00 | 100.00 | 4.3751 | 0.0035 | 0.0911 |
| GO:0002429 | immune response-activating cell surface receptor signaling pathway | 7 | 103 | 108 | 6.80 | 95.37 | 4.3740 | 0.0015 | 0.0482 |
| GO:0055082 | cellular chemical homeostasis | 16 | 376 | 379 | 4.26 | 99.21 | 4.3623 | 0.0000 | 0.0200 |
| GO:0023057 | negative regulation of signaling | 17 | 412 | 414 | 4.13 | 99.52 | 4.3549 | 0.0000 | 0.0200 |
| GO:0043549 | regulation of kinase activity | 18 | 450 | 454 | 4.00 | 99.12 | 4.3353 | 0.0000 | 0.0200 |
| GO:0006915 | apoptosis | 22 | 602 | 608 | 3.65 | 99.01 | 4.3335 | 0.0000 | 0.0200 |
| GO:0055080 | cation homeostasis | 14 | 310 | 312 | 4.52 | 99.36 | 4.3311 | 0.0010 | 0.0344 |
| GO:0034599 | cellular response to oxidative stress | 5 | 60 | 62 | 8.33 | 96.77 | 4.3069 | 0.0010 | 0.0344 |
| GO:0031331 | positive regulation of cellular catabolic process | 5 | 60 | 62 | 8.33 | 96.77 | 4.3069 | 0.0025 | 0.0712 |
| GO:0051130 | positive regulation of cellular component organization | 13 | 280 | 283 | 4.64 | 98.94 | 4.2869 | 0.0005 | 0.0200 |
| GO:0007167 | enzyme linked receptor protein signaling pathway | 21 | 569 | 570 | 3.69 | 99.82 | 4.2804 | 0.0000 | 0.0200 |
| GO:0009620 | response to fungus | 3 | 25 | 25 | 12.00 | 100.00 | 4.2734 | 0.0060 | 0.1345 |
| GO:0045840 | positive regulation of mitosis | 3 | 25 | 25 | 12.00 | 100.00 | 4.2734 | 0.0060 | 0.1345 |
| GO:0051785 | positive regulation of nuclear division | 3 | 25 | 25 | 12.00 | 100.00 | 4.2734 | 0.0060 | 0.1345 |
| GO:0030003 | cellular cation homeostasis | 13 | 281 | 283 | 4.63 | 99.29 | 4.2719 | 0.0005 | 0.0200 |
| GO:0042592 | homeostatic process | 25 | 731 | 738 | 3.42 | 99.05 | 4.2655 | 0.0000 | 0.0200 |
| GO:0012501 | programmed cell death | 22 | 612 | 618 | 3.59 | 99.03 | 4.2480 | 0.0000 | 0.0200 |
| GO:0002768 | immune response-regulating cell surface receptor signaling pathway | 7 | 107 | 112 | 6.54 | 95.54 | 4.2436 | 0.0015 | 0.0482 |
| GO:0050670 | regulation of lymphocyte proliferation | 7 | 107 | 108 | 6.54 | 99.07 | 4.2436 | 0.0015 | 0.0482 |
| GO:0019362 | pyridine nucleotide metabolic process | 4 | 42 | 42 | 9.52 | 100.00 | 4.2314 | 0.0030 | 0.0813 |
| GO:0032944 | regulation of mononuclear cell proliferation | 7 | 108 | 109 | 6.48 | 99.08 | 4.2121 | 0.0015 | 0.0482 |
| GO:2000112 | regulation of cellular macromolecule biosynthetic process | 54 | 2088 | 2154 | 2.59 | 96.94 | 4.2115 | 0.0000 | 0.0200 |
| GO:0006879 | cellular iron ion homeostasis | 5 | 62 | 63 | 8.06 | 98.41 | 4.2054 | 0.0035 | 0.0911 |
| GO:0050727 | regulation of inflammatory response | 7 | 109 | 110 | 6.42 | 99.09 | 4.1808 | 0.0010 | 0.0344 |
| GO:0051252 | regulation of RNA metabolic process | 51 | 1947 | 2012 | 2.62 | 96.77 | 4.1749 | 0.0000 | 0.0200 |
| GO:0006875 | cellular metal ion homeostasis | 12 | 255 | 257 | 4.71 | 99.22 | 4.1707 | 0.0000 | 0.0200 |
| GO:0002224 | toll-like receptor signaling pathway | 6 | 85 | 85 | 7.06 | 100.00 | 4.1694 | 0.0020 | 0.0604 |
| GO:0042306 | regulation of protein import into nucleus | 6 | 85 | 85 | 7.06 | 100.00 | 4.1694 | 0.0030 | 0.0813 |
| GO:0051338 | regulation of transferase activity | 18 | 466 | 470 | 3.86 | 99.15 | 4.1687 | 0.0000 | 0.0200 |
| GO:0000188 | inactivation of MAPK activity | 3 | 26 | 26 | 11.54 | 100.00 | 4.1661 | 0.0020 | 0.0604 |
| GO:0009409 | response to cold | 3 | 26 | 26 | 11.54 | 100.00 | 4.1661 | 0.0065 | 0.1432 |
| GO:0072524 | pyridine-containing compound metabolic process | 4 | 43 | 43 | 9.30 | 100.00 | 4.1630 | 0.0035 | 0.0911 |
| GO:0050818 | regulation of coagulation | 4 | 43 | 45 | 9.30 | 95.56 | 4.1630 | 0.0040 | 0.0989 |
| GO:0043086 | negative regulation of catalytic activity | 16 | 393 | 396 | 4.07 | 99.24 | 4.1610 | 0.0005 | 0.0200 |
| GO:0050864 | regulation of B cell activation | 5 | 63 | 64 | 7.94 | 98.44 | 4.1563 | 0.0015 | 0.0482 |
| GO:0034138 | toll-like receptor 3 signaling pathway | 5 | 63 | 63 | 7.94 | 100.00 | 4.1563 | 0.0030 | 0.0813 |
| GO:0050801 | ion homeostasis | 16 | 394 | 397 | 4.06 | 99.24 | 4.1495 | 0.0005 | 0.0200 |
| GO:0071822 | protein complex subunit organization | 20 | 545 | 550 | 3.67 | 99.09 | 4.1456 | 0.0000 | 0.0200 |
| GO:0051049 | regulation of transport | 23 | 665 | 671 | 3.46 | 99.11 | 4.1432 | 0.0005 | 0.0200 |
| GO:0048585 | negative regulation of response to stimulus | 18 | 469 | 471 | 3.84 | 99.58 | 4.1382 | 0.0000 | 0.0200 |
| GO:0045859 | regulation of protein kinase activity | 17 | 432 | 436 | 3.94 | 99.08 | 4.1341 | 0.0000 | 0.0200 |
| GO:0070663 | regulation of leukocyte proliferation | 7 | 111 | 112 | 6.31 | 99.11 | 4.1195 | 0.0025 | 0.0712 |
| GO:0045619 | regulation of lymphocyte differentiation | 5 | 64 | 65 | 7.81 | 98.46 | 4.1082 | 0.0020 | 0.0604 |
| GO:0006873 | cellular ion homeostasis | 15 | 362 | 365 | 4.14 | 99.18 | 4.1034 | 0.0005 | 0.0200 |
| GO:0045621 | positive regulation of lymphocyte differentiation | 4 | 44 | 45 | 9.09 | 97.78 | 4.0968 | 0.0030 | 0.0813 |
| GO:0042307 | positive regulation of protein import into nucleus | 4 | 44 | 44 | 9.09 | 100.00 | 4.0968 | 0.0040 | 0.0989 |
| GO:0022607 | cellular component assembly | 30 | 971 | 983 | 3.09 | 98.78 | 4.0881 | 0.0000 | 0.0200 |
| GO:0007568 | aging | 8 | 139 | 139 | 5.76 | 100.00 | 4.0819 | 0.0010 | 0.0344 |
| GO:0030154 | cell differentiation | 33 | 1108 | 1117 | 2.98 | 99.19 | 4.0733 | 0.0005 | 0.0200 |
| GO:0008285 | negative regulation of cell proliferation | 16 | 401 | 403 | 3.99 | 99.50 | 4.0700 | 0.0005 | 0.0200 |
| GO:0045088 | regulation of innate immune response | 9 | 168 | 169 | 5.36 | 99.41 | 4.0684 | 0.0005 | 0.0200 |
| GO:0002763 | positive regulation of myeloid leukocyte differentiation | 3 | 27 | 27 | 11.11 | 100.00 | 4.0643 | 0.0055 | 0.1260 |
| GO:0007006 | mitochondrial membrane organization | 3 | 27 | 27 | 11.11 | 100.00 | 4.0643 | 0.0055 | 0.1260 |
| GO:0007259 | JAK-STAT cascade | 3 | 27 | 27 | 11.11 | 100.00 | 4.0643 | 0.0055 | 0.1260 |
| GO:0002709 | regulation of T cell mediated immunity | 3 | 27 | 28 | 11.11 | 96.43 | 4.0643 | 0.0075 | 0.1589 |
| GO:0010883 | regulation of lipid storage | 3 | 27 | 27 | 11.11 | 100.00 | 4.0643 | 0.0075 | 0.1589 |
| GO:0002718 | regulation of cytokine production involved in immune response | 3 | 27 | 27 | 11.11 | 100.00 | 4.0643 | 0.0085 | 0.1730 |
| GO:0010676 | positive regulation of cellular carbohydrate metabolic process | 3 | 27 | 27 | 11.11 | 100.00 | 4.0643 | 0.0090 | 0.1813 |
| GO:0045913 | positive regulation of carbohydrate metabolic process | 3 | 27 | 27 | 11.11 | 100.00 | 4.0643 | 0.0090 | 0.1813 |
| GO:0048469 | cell maturation | 5 | 65 | 65 | 7.69 | 100.00 | 4.0611 | 0.0020 | 0.0604 |
| GO:0060337 | type I interferon-mediated signaling pathway | 5 | 65 | 65 | 7.69 | 100.00 | 4.0611 | 0.0020 | 0.0604 |
| GO:0071357 | cellular response to type I interferon | 5 | 65 | 65 | 7.69 | 100.00 | 4.0611 | 0.0020 | 0.0604 |
| GO:0002274 | myeloid leukocyte activation | 4 | 45 | 45 | 8.89 | 100.00 | 4.0325 | 0.0040 | 0.0989 |
| GO:0071219 | cellular response to molecule of bacterial origin | 4 | 45 | 45 | 8.89 | 100.00 | 4.0325 | 0.0040 | 0.0989 |
| GO:0045639 | positive regulation of myeloid cell differentiation | 4 | 45 | 45 | 8.89 | 100.00 | 4.0325 | 0.0060 | 0.1345 |
| GO:0010638 | positive regulation of organelle organization | 7 | 114 | 116 | 6.14 | 98.28 | 4.0302 | 0.0015 | 0.0482 |
| GO:0002221 | pattern recognition receptor signaling pathway | 6 | 89 | 89 | 6.74 | 100.00 | 4.0221 | 0.0025 | 0.0712 |
| GO:0034340 | response to type I interferon | 5 | 66 | 66 | 7.58 | 100.00 | 4.0150 | 0.0020 | 0.0604 |
| GO:0060333 | interferon-gamma-mediated signaling pathway | 5 | 66 | 67 | 7.58 | 98.51 | 4.0150 | 0.0020 | 0.0604 |
| GO:0055072 | iron ion homeostasis | 5 | 66 | 67 | 7.58 | 98.51 | 4.0150 | 0.0035 | 0.0911 |
| GO:0050731 | positive regulation of peptidyl-tyrosine phosphorylation | 5 | 66 | 67 | 7.58 | 98.51 | 4.0150 | 0.0045 | 0.1080 |
| GO:0010646 | regulation of cell communication | 30 | 984 | 990 | 3.05 | 99.39 | 4.0095 | 0.0000 | 0.0200 |
| GO:0071900 | regulation of protein serine/threonine kinase activity | 12 | 266 | 269 | 4.51 | 98.88 | 4.0002 | 0.0010 | 0.0344 |
| GO:0050790 | regulation of catalytic activity | 32 | 1078 | 1095 | 2.97 | 98.45 | 3.9867 | 0.0000 | 0.0200 |
| GO:0051171 | regulation of nitrogen compound metabolic process | 59 | 2408 | 2481 | 2.45 | 97.06 | 3.9844 | 0.0000 | 0.0200 |
| GO:0043933 | macromolecular complex subunit organization | 25 | 771 | 782 | 3.24 | 98.59 | 3.9749 | 0.0000 | 0.0200 |
| GO:0006469 | negative regulation of protein kinase activity | 7 | 116 | 116 | 6.03 | 100.00 | 3.9724 | 0.0010 | 0.0344 |
| GO:0034764 | positive regulation of transmembrane transport | 4 | 46 | 46 | 8.70 | 100.00 | 3.9702 | 0.0040 | 0.0989 |
| GO:0051897 | positive regulation of protein kinase B signaling cascade | 3 | 28 | 29 | 10.71 | 96.55 | 3.9676 | 0.0050 | 0.1166 |
| GO:0015850 | organic alcohol transport | 3 | 28 | 29 | 10.71 | 96.55 | 3.9676 | 0.0060 | 0.1345 |
| GO:0035821 | modification of morphology or physiology of other organism | 3 | 28 | 28 | 10.71 | 100.00 | 3.9676 | 0.0080 | 0.1662 |
| GO:0051101 | regulation of DNA binding | 3 | 28 | 28 | 10.71 | 100.00 | 3.9676 | 0.0080 | 0.1662 |
| GO:0051817 | modification of morphology or physiology of other organism involved in symbiotic interaction | 3 | 28 | 28 | 10.71 | 100.00 | 3.9676 | 0.0080 | 0.1662 |
| GO:0023051 | regulation of signaling | 39 | 1410 | 1421 | 2.77 | 99.23 | 3.9601 | 0.0000 | 0.0200 |
| GO:0002758 | innate immune response-activating signal transduction | 6 | 91 | 92 | 6.59 | 98.91 | 3.9517 | 0.0025 | 0.0712 |
| GO:0000302 | response to reactive oxygen species | 6 | 91 | 92 | 6.59 | 98.91 | 3.9517 | 0.0030 | 0.0813 |
| GO:0010648 | negative regulation of cell communication | 16 | 413 | 415 | 3.87 | 99.52 | 3.9374 | 0.0005 | 0.0200 |
| GO:0035556 | intracellular signal transduction | 33 | 1134 | 1142 | 2.91 | 99.30 | 3.9302 | 0.0005 | 0.0200 |
| GO:0031324 | negative regulation of cellular metabolic process | 23 | 693 | 697 | 3.32 | 99.43 | 3.9269 | 0.0015 | 0.0482 |
| GO:0002703 | regulation of leukocyte mediated immunity | 5 | 68 | 69 | 7.35 | 98.55 | 3.9255 | 0.0010 | 0.0344 |
| GO:0007565 | female pregnancy | 5 | 68 | 69 | 7.35 | 98.55 | 3.9255 | 0.0045 | 0.1080 |
| GO:0032386 | regulation of intracellular transport | 7 | 118 | 118 | 5.93 | 100.00 | 3.9158 | 0.0035 | 0.0911 |
| GO:0034621 | cellular macromolecular complex subunit organization | 17 | 453 | 461 | 3.75 | 98.26 | 3.9152 | 0.0000 | 0.0200 |
| GO:0002263 | cell activation involved in immune response | 4 | 47 | 47 | 8.51 | 100.00 | 3.9097 | 0.0035 | 0.0911 |
| GO:0002366 | leukocyte activation involved in immune response | 4 | 47 | 47 | 8.51 | 100.00 | 3.9097 | 0.0035 | 0.0911 |
| GO:0007155 | cell adhesion | 23 | 696 | 703 | 3.30 | 99.00 | 3.9043 | 0.0000 | 0.0200 |
| GO:0022610 | biological adhesion | 23 | 696 | 703 | 3.30 | 99.00 | 3.9043 | 0.0000 | 0.0200 |
| GO:0019219 | regulation of nucleobase, nucleoside, nucleotide and nucleic acid metabolic process | 57 | 2330 | 2402 | 2.45 | 97.00 | 3.8929 | 0.0000 | 0.0200 |
| GO:0023056 | positive regulation of signaling | 18 | 495 | 498 | 3.64 | 99.40 | 3.8835 | 0.0010 | 0.0344 |
| GO:0002218 | activation of innate immune response | 6 | 93 | 94 | 6.45 | 98.94 | 3.8834 | 0.0030 | 0.0813 |
| GO:0008637 | apoptotic mitochondrial changes | 3 | 29 | 29 | 10.34 | 100.00 | 3.8756 | 0.0075 | 0.1589 |
| GO:0043154 | negative regulation of caspase activity | 3 | 29 | 29 | 10.34 | 100.00 | 3.8756 | 0.0080 | 0.1662 |
| GO:0033138 | positive regulation of peptidyl-serine phosphorylation | 3 | 29 | 29 | 10.34 | 100.00 | 3.8756 | 0.0100 | 0.1989 |
| GO:0009968 | negative regulation of signal transduction | 15 | 382 | 384 | 3.93 | 99.48 | 3.8681 | 0.0005 | 0.0200 |
| GO:0045089 | positive regulation of innate immune response | 7 | 120 | 121 | 5.83 | 99.17 | 3.8604 | 0.0025 | 0.0712 |
| GO:0022603 | regulation of anatomical structure morphogenesis | 14 | 348 | 351 | 4.02 | 99.15 | 3.8362 | 0.0000 | 0.0200 |
| GO:0019725 | cellular homeostasis | 17 | 463 | 466 | 3.67 | 99.36 | 3.8152 | 0.0000 | 0.0200 |
| GO:0051726 | regulation of cell cycle | 20 | 583 | 583 | 3.43 | 100.00 | 3.8134 | 0.0000 | 0.0200 |
| GO:0033673 | negative regulation of kinase activity | 7 | 122 | 122 | 5.74 | 100.00 | 3.8062 | 0.0010 | 0.0344 |
| GO:0071901 | negative regulation of protein serine/threonine kinase activity | 5 | 71 | 71 | 7.04 | 100.00 | 3.7976 | 0.0000 | 0.0200 |
| GO:0045807 | positive regulation of endocytosis | 4 | 49 | 50 | 8.16 | 98.00 | 3.7936 | 0.0045 | 0.1080 |
| GO:0043524 | negative regulation of neuron apoptosis | 4 | 49 | 50 | 8.16 | 98.00 | 3.7936 | 0.0050 | 0.1166 |
| GO:0051128 | regulation of cellular component organization | 25 | 798 | 804 | 3.13 | 99.25 | 3.7884 | 0.0005 | 0.0200 |
| GO:0002698 | negative regulation of immune effector process | 3 | 30 | 30 | 10.00 | 100.00 | 3.7878 | 0.0060 | 0.1345 |
| GO:0022406 | membrane docking | 3 | 30 | 30 | 10.00 | 100.00 | 3.7878 | 0.0060 | 0.1345 |
| GO:0045670 | regulation of osteoclast differentiation | 3 | 30 | 30 | 10.00 | 100.00 | 3.7878 | 0.0075 | 0.1589 |
| GO:0000902 | cell morphogenesis | 6 | 96 | 99 | 6.25 | 96.97 | 3.7843 | 0.0020 | 0.0604 |
| GO:0009966 | regulation of signal transduction | 34 | 1210 | 1220 | 2.81 | 99.18 | 3.7748 | 0.0005 | 0.0200 |
| GO:0071216 | cellular response to biotic stimulus | 5 | 72 | 72 | 6.94 | 100.00 | 3.7565 | 0.0030 | 0.0813 |
| GO:0071346 | cellular response to interferon-gamma | 5 | 72 | 73 | 6.94 | 98.63 | 3.7565 | 0.0030 | 0.0813 |
| GO:0051789 | response to protein stimulus | 7 | 124 | 126 | 5.65 | 98.41 | 3.7532 | 0.0010 | 0.0344 |
| GO:0002697 | regulation of immune effector process | 8 | 153 | 154 | 5.23 | 99.35 | 3.7507 | 0.0030 | 0.0813 |
| GO:0045860 | positive regulation of protein kinase activity | 12 | 284 | 287 | 4.23 | 98.95 | 3.7393 | 0.0005 | 0.0200 |
| GO:0030162 | regulation of proteolysis | 7 | 125 | 127 | 5.60 | 98.43 | 3.7271 | 0.0015 | 0.0482 |
| GO:0032989 | cellular component morphogenesis | 10 | 218 | 221 | 4.59 | 98.64 | 3.7082 | 0.0005 | 0.0200 |
| GO:0006355 | regulation of transcription, DNA-dependent | 47 | 1865 | 1930 | 2.52 | 96.63 | 3.7046 | 0.0000 | 0.0200 |
| GO:0035303 | regulation of dephosphorylation | 3 | 31 | 33 | 9.68 | 93.94 | 3.7040 | 0.0070 | 0.1513 |
| GO:0042401 | cellular biogenic amine biosynthetic process | 3 | 31 | 31 | 9.68 | 100.00 | 3.7040 | 0.0110 | 0.2130 |
| GO:0002690 | positive regulation of leukocyte chemotaxis | 3 | 31 | 31 | 9.68 | 100.00 | 3.7040 | 0.0125 | 0.2308 |
| GO:0033157 | regulation of intracellular protein transport | 6 | 99 | 99 | 6.06 | 100.00 | 3.6892 | 0.0040 | 0.0989 |
| GO:0043410 | positive regulation of MAPKKK cascade | 6 | 99 | 101 | 6.06 | 98.02 | 3.6892 | 0.0040 | 0.0989 |
| GO:0042102 | positive regulation of T cell proliferation | 4 | 51 | 51 | 7.84 | 100.00 | 3.6838 | 0.0040 | 0.0989 |
| GO:0046824 | positive regulation of nucleocytoplasmic transport | 4 | 51 | 51 | 7.84 | 100.00 | 3.6838 | 0.0045 | 0.1080 |
| GO:0006733 | oxidoreduction coenzyme metabolic process | 4 | 51 | 51 | 7.84 | 100.00 | 3.6838 | 0.0070 | 0.1513 |
| GO:0042446 | hormone biosynthetic process | 5 | 74 | 74 | 6.76 | 100.00 | 3.6766 | 0.0035 | 0.0911 |
| GO:0007186 | G-protein coupled receptor protein signaling pathway | 18 | 519 | 524 | 3.47 | 99.05 | 3.6622 | 0.0000 | 0.0200 |
| GO:0044237 | cellular metabolic process | 117 | 5838 | 5932 | 2.00 | 98.42 | 3.6601 | 0.0015 | 0.0482 |
| GO:0071844 | cellular component assembly at cellular level | 22 | 688 | 698 | 3.20 | 98.57 | 3.6474 | 0.0010 | 0.0344 |
| GO:0007584 | response to nutrient | 9 | 189 | 190 | 4.76 | 99.47 | 3.6468 | 0.0015 | 0.0482 |
| GO:0006690 | icosanoid metabolic process | 4 | 52 | 52 | 7.69 | 100.00 | 3.6310 | 0.0055 | 0.1260 |
| GO:0009895 | negative regulation of catabolic process | 4 | 52 | 52 | 7.69 | 100.00 | 3.6310 | 0.0055 | 0.1260 |
| GO:0051348 | negative regulation of transferase activity | 7 | 129 | 129 | 5.43 | 100.00 | 3.6253 | 0.0020 | 0.0604 |
| GO:0001704 | formation of primary germ layer | 3 | 32 | 32 | 9.38 | 100.00 | 3.6237 | 0.0065 | 0.1432 |
| GO:0002285 | lymphocyte activation involved in immune response | 3 | 32 | 32 | 9.38 | 100.00 | 3.6237 | 0.0105 | 0.2062 |
| GO:0046635 | positive regulation of alpha-beta T cell activation | 3 | 32 | 32 | 9.38 | 100.00 | 3.6237 | 0.0105 | 0.2062 |
| GO:0043255 | regulation of carbohydrate biosynthetic process | 3 | 32 | 33 | 9.38 | 96.97 | 3.6237 | 0.0125 | 0.2308 |
| GO:0048146 | positive regulation of fibroblast proliferation | 3 | 32 | 33 | 9.38 | 96.97 | 3.6237 | 0.0125 | 0.2308 |
| GO:0010740 | positive regulation of intracellular protein kinase cascade | 11 | 258 | 261 | 4.26 | 98.85 | 3.6118 | 0.0020 | 0.0604 |
| GO:0051704 | multi-organism process | 22 | 693 | 700 | 3.17 | 99.00 | 3.6107 | 0.0000 | 0.0200 |
| GO:0043405 | regulation of MAP kinase activity | 9 | 191 | 194 | 4.71 | 98.45 | 3.6097 | 0.0025 | 0.0712 |
| GO:0033674 | positive regulation of kinase activity | 12 | 294 | 297 | 4.08 | 98.99 | 3.6030 | 0.0005 | 0.0200 |
| GO:0051346 | negative regulation of hydrolase activity | 7 | 130 | 133 | 5.38 | 97.74 | 3.6004 | 0.0040 | 0.0989 |
| GO:0009636 | response to toxin | 5 | 76 | 77 | 6.58 | 98.70 | 3.5995 | 0.0035 | 0.0911 |
| GO:0010212 | response to ionizing radiation | 5 | 76 | 77 | 6.58 | 98.70 | 3.5995 | 0.0065 | 0.1432 |
| GO:0000041 | transition metal ion transport | 6 | 102 | 103 | 5.88 | 99.03 | 3.5978 | 0.0065 | 0.1432 |
| GO:0006109 | regulation of carbohydrate metabolic process | 4 | 53 | 54 | 7.55 | 98.15 | 3.5796 | 0.0045 | 0.1080 |
| GO:0010675 | regulation of cellular carbohydrate metabolic process | 4 | 53 | 54 | 7.55 | 98.15 | 3.5796 | 0.0045 | 0.1080 |
| GO:0090316 | positive regulation of intracellular protein transport | 4 | 53 | 53 | 7.55 | 100.00 | 3.5796 | 0.0075 | 0.1589 |
| GO:0043433 | negative regulation of sequence-specific DNA binding transcription factor activity | 5 | 77 | 77 | 6.49 | 100.00 | 3.5619 | 0.0045 | 0.1080 |
| GO:0050852 | T cell receptor signaling pathway | 5 | 77 | 81 | 6.49 | 95.06 | 3.5619 | 0.0065 | 0.1432 |
| GO:0045935 | positive regulation of nucleobase, nucleoside, nucleotide and nucleic acid metabolic process | 19 | 572 | 575 | 3.32 | 99.48 | 3.5601 | 0.0020 | 0.0604 |
| GO:0006917 | induction of apoptosis | 13 | 335 | 335 | 3.88 | 100.00 | 3.5477 | 0.0015 | 0.0482 |
| GO:0045740 | positive regulation of DNA replication | 3 | 33 | 33 | 9.09 | 100.00 | 3.5468 | 0.0080 | 0.1662 |
| GO:0044093 | positive regulation of molecular function | 23 | 747 | 751 | 3.08 | 99.47 | 3.5369 | 0.0000 | 0.0200 |
| GO:0043407 | negative regulation of MAP kinase activity | 4 | 54 | 54 | 7.41 | 100.00 | 3.5294 | 0.0030 | 0.0813 |
| GO:0021700 | developmental maturation | 5 | 78 | 78 | 6.41 | 100.00 | 3.5250 | 0.0035 | 0.0911 |
| GO:0006959 | humoral immune response | 5 | 78 | 98 | 6.41 | 79.59 | 3.5250 | 0.0060 | 0.1345 |
| GO:0012502 | induction of programmed cell death | 13 | 337 | 337 | 3.86 | 100.00 | 3.5237 | 0.0015 | 0.0482 |
| GO:0045087 | innate immune response | 10 | 230 | 250 | 4.35 | 92.00 | 3.5122 | 0.0020 | 0.0604 |
| GO:0072507 | divalent inorganic cation homeostasis | 9 | 197 | 198 | 4.57 | 99.49 | 3.5014 | 0.0025 | 0.0712 |
| GO:0008629 | induction of apoptosis by intracellular signals | 5 | 79 | 79 | 6.33 | 100.00 | 3.4887 | 0.0040 | 0.0989 |
| GO:0051050 | positive regulation of transport | 12 | 303 | 306 | 3.96 | 99.02 | 3.4851 | 0.0010 | 0.0344 |
| GO:0051347 | positive regulation of transferase activity | 12 | 303 | 306 | 3.96 | 99.02 | 3.4851 | 0.0010 | 0.0344 |
| GO:0033559 | unsaturated fatty acid metabolic process | 4 | 55 | 55 | 7.27 | 100.00 | 3.4805 | 0.0070 | 0.1513 |
| GO:0045580 | regulation of T cell differentiation | 4 | 55 | 56 | 7.27 | 98.21 | 3.4805 | 0.0080 | 0.1662 |
| GO:0043627 | response to estrogen stimulus | 7 | 135 | 135 | 5.19 | 100.00 | 3.4799 | 0.0040 | 0.0989 |
| GO:0046651 | lymphocyte proliferation | 3 | 34 | 35 | 8.82 | 97.14 | 3.4730 | 0.0135 | 0.2308 |
| GO:0007169 | transmembrane receptor protein tyrosine kinase signaling pathway | 16 | 462 | 463 | 3.46 | 99.78 | 3.4404 | 0.0000 | 0.0200 |
| GO:0032388 | positive regulation of intracellular transport | 4 | 56 | 56 | 7.14 | 100.00 | 3.4327 | 0.0085 | 0.1730 |
| GO:0008283 | cell proliferation | 15 | 424 | 430 | 3.54 | 98.60 | 3.4189 | 0.0020 | 0.0604 |
| GO:0031099 | regeneration | 5 | 81 | 81 | 6.17 | 100.00 | 3.4178 | 0.0035 | 0.0911 |
| GO:0030100 | regulation of endocytosis | 5 | 81 | 82 | 6.17 | 98.78 | 3.4178 | 0.0050 | 0.1166 |
| GO:0009896 | positive regulation of catabolic process | 5 | 81 | 83 | 6.17 | 97.59 | 3.4178 | 0.0070 | 0.1513 |
| GO:0019216 | regulation of lipid metabolic process | 7 | 138 | 139 | 5.07 | 99.28 | 3.4102 | 0.0035 | 0.0911 |
| GO:0032663 | regulation of interleukin-2 production | 3 | 35 | 35 | 8.57 | 100.00 | 3.4020 | 0.0175 | 0.2577 |
| GO:0016043 | cellular component organization | 61 | 2694 | 2721 | 2.26 | 99.01 | 3.3990 | 0.0010 | 0.0344 |
| GO:0010038 | response to metal ion | 8 | 170 | 172 | 4.71 | 98.84 | 3.3968 | 0.0035 | 0.0911 |
| GO:0006826 | iron ion transport | 4 | 57 | 58 | 7.02 | 98.28 | 3.3860 | 0.0075 | 0.1589 |
| GO:0007229 | integrin-mediated signaling pathway | 4 | 57 | 57 | 7.02 | 100.00 | 3.3860 | 0.0090 | 0.1813 |
| GO:0032355 | response to estradiol stimulus | 5 | 82 | 82 | 6.10 | 100.00 | 3.3832 | 0.0070 | 0.1513 |
| GO:0008152 | metabolic process | 129 | 6713 | 6851 | 1.92 | 97.99 | 3.3797 | 0.0050 | 0.1166 |
| GO:2000241 | regulation of reproductive process | 6 | 110 | 110 | 5.45 | 100.00 | 3.3700 | 0.0050 | 0.1166 |
| GO:0006351 | transcription, DNA-dependent | 23 | 773 | 781 | 2.98 | 98.98 | 3.3606 | 0.0015 | 0.0482 |
| GO:0043085 | positive regulation of catalytic activity | 20 | 642 | 646 | 3.12 | 99.38 | 3.3449 | 0.0025 | 0.0712 |
| GO:0051091 | positive regulation of sequence-specific DNA binding transcription factor activity | 6 | 111 | 112 | 5.41 | 99.11 | 3.3430 | 0.0025 | 0.0712 |
| GO:0002706 | regulation of lymphocyte mediated immunity | 4 | 58 | 59 | 6.90 | 98.31 | 3.3404 | 0.0090 | 0.1813 |
| GO:0060326 | cell chemotaxis | 4 | 58 | 59 | 6.90 | 98.31 | 3.3404 | 0.0110 | 0.2130 |
| GO:0006833 | water transport | 3 | 36 | 37 | 8.33 | 97.30 | 3.3337 | 0.0125 | 0.2308 |
| GO:0002824 | positive regulation of adaptive immune response based on somatic recombination of immune receptors built from immunoglobulin superfamily domains | 3 | 36 | 37 | 8.33 | 97.30 | 3.3337 | 0.0135 | 0.2308 |
| GO:0051952 | regulation of amine transport | 3 | 36 | 37 | 8.33 | 97.30 | 3.3337 | 0.0140 | 0.2330 |
| GO:0032943 | mononuclear cell proliferation | 3 | 36 | 37 | 8.33 | 97.30 | 3.3337 | 0.0155 | 0.2410 |
| GO:0033135 | regulation of peptidyl-serine phosphorylation | 3 | 36 | 36 | 8.33 | 100.00 | 3.3337 | 0.0155 | 0.2410 |
| GO:0002688 | regulation of leukocyte chemotaxis | 3 | 36 | 36 | 8.33 | 100.00 | 3.3337 | 0.0175 | 0.2577 |
| GO:0032880 | regulation of protein localization | 9 | 207 | 209 | 4.35 | 99.04 | 3.3297 | 0.0035 | 0.0911 |
| GO:0030182 | neuron differentiation | 7 | 142 | 142 | 4.93 | 100.00 | 3.3202 | 0.0080 | 0.1662 |
| GO:0043281 | regulation of caspase activity | 6 | 112 | 113 | 5.36 | 99.12 | 3.3163 | 0.0075 | 0.1589 |
| GO:0045471 | response to ethanol | 5 | 84 | 85 | 5.95 | 98.82 | 3.3157 | 0.0085 | 0.1730 |
| GO:0071840 | cellular component organization or biogenesis | 61 | 2725 | 2752 | 2.24 | 99.02 | 3.3026 | 0.0010 | 0.0344 |
| GO:0016311 | dephosphorylation | 7 | 143 | 146 | 4.90 | 97.95 | 3.2983 | 0.0050 | 0.1166 |
| GO:0007018 | microtubule-based movement | 6 | 113 | 119 | 5.31 | 94.96 | 3.2900 | 0.0065 | 0.1432 |
| GO:0046456 | icosanoid biosynthetic process | 3 | 37 | 37 | 8.11 | 100.00 | 3.2680 | 0.0110 | 0.2130 |
| GO:0070661 | leukocyte proliferation | 3 | 37 | 38 | 8.11 | 97.37 | 3.2680 | 0.0160 | 0.2428 |
| GO:0050730 | regulation of peptidyl-tyrosine phosphorylation | 5 | 86 | 87 | 5.81 | 98.85 | 3.2501 | 0.0115 | 0.2205 |
| GO:0010605 | negative regulation of macromolecule metabolic process | 21 | 701 | 705 | 3.00 | 99.43 | 3.2380 | 0.0040 | 0.0989 |
| GO:0051254 | positive regulation of RNA metabolic process | 16 | 486 | 488 | 3.29 | 99.59 | 3.2193 | 0.0030 | 0.0813 |
| GO:0060341 | regulation of cellular localization | 14 | 404 | 408 | 3.47 | 99.02 | 3.2152 | 0.0030 | 0.0813 |
| GO:0043279 | response to alkaloid | 4 | 61 | 61 | 6.56 | 100.00 | 3.2096 | 0.0100 | 0.1989 |
| GO:0006941 | striated muscle contraction | 3 | 38 | 39 | 7.89 | 97.44 | 3.2046 | 0.0155 | 0.2410 |
| GO:0042044 | fluid transport | 3 | 38 | 39 | 7.89 | 97.44 | 3.2046 | 0.0155 | 0.2410 |
| GO:0032768 | regulation of monooxygenase activity | 3 | 38 | 38 | 7.89 | 100.00 | 3.2046 | 0.0205 | 0.2853 |
| GO:0051098 | regulation of binding | 5 | 88 | 89 | 5.68 | 98.88 | 3.1866 | 0.0085 | 0.1730 |
| GO:0051179 | localization | 9 | 217 | 217 | 4.15 | 100.00 | 3.1680 | 0.0040 | 0.0989 |
| GO:0007179 | transforming growth factor beta receptor signaling pathway | 4 | 62 | 62 | 6.45 | 100.00 | 3.1679 | 0.0165 | 0.2483 |
| GO:0010647 | positive regulation of cell communication | 16 | 492 | 495 | 3.25 | 99.39 | 3.1660 | 0.0060 | 0.1345 |
| GO:0045596 | negative regulation of cell differentiation | 10 | 254 | 256 | 3.94 | 99.22 | 3.1556 | 0.0045 | 0.1080 |
| GO:0051896 | regulation of protein kinase B signaling cascade | 3 | 39 | 40 | 7.69 | 97.50 | 3.1434 | 0.0140 | 0.2330 |
| GO:0019722 | calcium-mediated signaling | 3 | 39 | 39 | 7.69 | 100.00 | 3.1434 | 0.0200 | 0.2823 |
| GO:0000904 | cell morphogenesis involved in differentiation | 4 | 63 | 64 | 6.35 | 98.44 | 3.1270 | 0.0145 | 0.2330 |
| GO:0009411 | response to UV | 4 | 63 | 63 | 6.35 | 100.00 | 3.1270 | 0.0145 | 0.2330 |
| GO:0032501 | multicellular organismal process | 64 | 2962 | 3038 | 2.16 | 97.50 | 3.0921 | 0.0020 | 0.0604 |
| GO:0006636 | unsaturated fatty acid biosynthetic process | 3 | 40 | 40 | 7.50 | 100.00 | 3.0842 | 0.0135 | 0.2308 |
| GO:0045582 | positive regulation of T cell differentiation | 3 | 40 | 41 | 7.50 | 97.56 | 3.0842 | 0.0190 | 0.2740 |
| GO:0046634 | regulation of alpha-beta T cell activation | 3 | 40 | 40 | 7.50 | 100.00 | 3.0842 | 0.0195 | 0.2789 |
| GO:0051149 | positive regulation of muscle cell differentiation | 3 | 40 | 40 | 7.50 | 100.00 | 3.0842 | 0.0215 | 0.2951 |
| GO:0045786 | negative regulation of cell cycle | 9 | 223 | 223 | 4.04 | 100.00 | 3.0753 | 0.0050 | 0.1166 |
| GO:0072503 | cellular divalent inorganic cation homeostasis | 8 | 189 | 190 | 4.23 | 99.47 | 3.0503 | 0.0040 | 0.0989 |
| GO:0031294 | lymphocyte costimulation | 4 | 65 | 69 | 6.15 | 94.20 | 3.0477 | 0.0160 | 0.2428 |
| GO:0031295 | T cell costimulation | 4 | 65 | 69 | 6.15 | 94.20 | 3.0477 | 0.0160 | 0.2428 |
| GO:0006836 | neurotransmitter transport | 5 | 93 | 94 | 5.38 | 98.94 | 3.0354 | 0.0135 | 0.2308 |
| GO:0071260 | cellular response to mechanical stimulus | 3 | 41 | 41 | 7.32 | 100.00 | 3.0270 | 0.0215 | 0.2951 |
| GO:0055074 | calcium ion homeostasis | 8 | 191 | 192 | 4.19 | 99.48 | 3.0164 | 0.0050 | 0.1166 |
| GO:0071214 | cellular response to abiotic stimulus | 4 | 66 | 67 | 6.06 | 98.51 | 3.0093 | 0.0135 | 0.2308 |
| GO:0051222 | positive regulation of protein transport | 5 | 94 | 96 | 5.32 | 97.92 | 3.0065 | 0.0115 | 0.2205 |
| GO:0006334 | nucleosome assembly | 5 | 94 | 99 | 5.32 | 94.95 | 3.0065 | 0.0155 | 0.2410 |
| GO:0009967 | positive regulation of signal transduction | 15 | 469 | 472 | 3.20 | 99.36 | 2.9928 | 0.0080 | 0.1662 |
| GO:0008217 | regulation of blood pressure | 5 | 95 | 95 | 5.26 | 100.00 | 2.9779 | 0.0105 | 0.2062 |
| GO:0051147 | regulation of muscle cell differentiation | 4 | 67 | 68 | 5.97 | 98.53 | 2.9716 | 0.0150 | 0.2378 |
| GO:0042542 | response to hydrogen peroxide | 4 | 67 | 68 | 5.97 | 98.53 | 2.9716 | 0.0175 | 0.2577 |
| GO:0007588 | excretion | 3 | 42 | 42 | 7.14 | 100.00 | 2.9716 | 0.0190 | 0.2740 |
| GO:0007569 | cell aging | 3 | 42 | 42 | 7.14 | 100.00 | 2.9716 | 0.0195 | 0.2789 |
| GO:0043112 | receptor metabolic process | 3 | 42 | 42 | 7.14 | 100.00 | 2.9716 | 0.0215 | 0.2951 |
| GO:0009187 | cyclic nucleotide metabolic process | 3 | 42 | 42 | 7.14 | 100.00 | 2.9716 | 0.0225 | 0.3049 |
| GO:0048145 | regulation of fibroblast proliferation | 3 | 42 | 43 | 7.14 | 97.67 | 2.9716 | 0.0240 | 0.3169 |
| GO:0032368 | regulation of lipid transport | 3 | 42 | 44 | 7.14 | 95.45 | 2.9716 | 0.0275 | 0.3416 |
| GO:0030030 | cell projection organization | 11 | 308 | 310 | 3.57 | 99.35 | 2.9525 | 0.0030 | 0.0813 |
| GO:0071902 | positive regulation of protein serine/threonine kinase activity | 7 | 160 | 163 | 4.38 | 98.16 | 2.9515 | 0.0115 | 0.2205 |
| GO:0051093 | negative regulation of developmental process | 11 | 309 | 311 | 3.56 | 99.36 | 2.9407 | 0.0060 | 0.1345 |
| GO:0060191 | regulation of lipase activity | 6 | 128 | 129 | 4.69 | 99.22 | 2.9268 | 0.0110 | 0.2130 |
| GO:0007005 | mitochondrion organization | 5 | 97 | 99 | 5.15 | 97.98 | 2.9218 | 0.0140 | 0.2330 |
| GO:0045995 | regulation of embryonic development | 3 | 43 | 43 | 6.98 | 100.00 | 2.9179 | 0.0220 | 0.3001 |
| GO:0032774 | RNA biosynthetic process | 24 | 894 | 903 | 2.68 | 99.00 | 2.8990 | 0.0030 | 0.0813 |
| GO:0045765 | regulation of angiogenesis | 5 | 98 | 99 | 5.10 | 98.99 | 2.8944 | 0.0130 | 0.2308 |
| GO:0016044 | cellular membrane organization | 13 | 395 | 397 | 3.29 | 99.50 | 2.8927 | 0.0060 | 0.1345 |
| GO:0006470 | protein dephosphorylation | 6 | 130 | 133 | 4.62 | 97.74 | 2.8825 | 0.0115 | 0.2205 |
| GO:0045944 | positive regulation of transcription from RNA polymerase II promoter | 11 | 314 | 316 | 3.50 | 99.37 | 2.8822 | 0.0080 | 0.1662 |
| GO:0061024 | membrane organization | 13 | 397 | 399 | 3.27 | 99.50 | 2.8729 | 0.0060 | 0.1345 |
| GO:0051781 | positive regulation of cell division | 3 | 44 | 44 | 6.82 | 100.00 | 2.8658 | 0.0290 | 0.3512 |
| GO:0009266 | response to temperature stimulus | 5 | 100 | 100 | 5.00 | 100.00 | 2.8405 | 0.0190 | 0.2740 |
| GO:0006887 | exocytosis | 7 | 166 | 169 | 4.22 | 98.22 | 2.8399 | 0.0105 | 0.2062 |
| GO:0045893 | positive regulation of transcription, DNA-dependent | 14 | 444 | 446 | 3.15 | 99.55 | 2.8311 | 0.0075 | 0.1589 |
| GO:0006886 | intracellular protein transport | 12 | 361 | 364 | 3.32 | 99.18 | 2.8142 | 0.0045 | 0.1080 |
| GO:0042113 | B cell activation | 4 | 72 | 72 | 5.56 | 100.00 | 2.7935 | 0.0230 | 0.3089 |
| GO:0044260 | cellular macromolecule metabolic process | 81 | 4060 | 4132 | 2.00 | 98.26 | 2.7838 | 0.0110 | 0.2130 |
| GO:0045862 | positive regulation of proteolysis | 3 | 46 | 47 | 6.52 | 97.87 | 2.7662 | 0.0295 | 0.3545 |
| GO:0030595 | leukocyte chemotaxis | 3 | 46 | 47 | 6.52 | 97.87 | 2.7662 | 0.0300 | 0.3577 |
| GO:0050921 | positive regulation of chemotaxis | 3 | 46 | 46 | 6.52 | 100.00 | 2.7662 | 0.0330 | 0.3810 |
| GO:0042129 | regulation of T cell proliferation | 4 | 73 | 73 | 5.48 | 100.00 | 2.7597 | 0.0225 | 0.3049 |
| GO:0007173 | epidermal growth factor receptor signaling pathway | 4 | 73 | 73 | 5.48 | 100.00 | 2.7597 | 0.0260 | 0.3350 |
| GO:0031669 | cellular response to nutrient levels | 4 | 73 | 73 | 5.48 | 100.00 | 2.7597 | 0.0270 | 0.3397 |
| GO:0060627 | regulation of vesicle-mediated transport | 6 | 136 | 137 | 4.41 | 99.27 | 2.7544 | 0.0135 | 0.2308 |
| GO:0010627 | regulation of intracellular protein kinase cascade | 12 | 368 | 371 | 3.26 | 99.19 | 2.7420 | 0.0100 | 0.1989 |
| GO:0000187 | activation of MAPK activity | 5 | 104 | 107 | 4.81 | 97.20 | 2.7366 | 0.0240 | 0.3169 |
| GO:0034728 | nucleosome organization | 5 | 104 | 109 | 4.81 | 95.41 | 2.7366 | 0.0240 | 0.3169 |
| GO:0065004 | protein-DNA complex assembly | 5 | 104 | 109 | 4.81 | 95.41 | 2.7366 | 0.0240 | 0.3169 |
| GO:0045785 | positive regulation of cell adhesion | 4 | 74 | 76 | 5.41 | 97.37 | 2.7266 | 0.0260 | 0.3350 |
| GO:0051641 | cellular localization | 6 | 138 | 138 | 4.35 | 100.00 | 2.7132 | 0.0185 | 0.2687 |
| GO:0010628 | positive regulation of gene expression | 15 | 504 | 506 | 2.98 | 99.60 | 2.6930 | 0.0080 | 0.1662 |
| GO:0090068 | positive regulation of cell cycle process | 5 | 106 | 106 | 4.72 | 100.00 | 2.6866 | 0.0220 | 0.3001 |
| GO:0006006 | glucose metabolic process | 6 | 140 | 142 | 4.29 | 98.59 | 2.6728 | 0.0215 | 0.2951 |
| GO:0033993 | response to lipid | 3 | 48 | 48 | 6.25 | 100.00 | 2.6721 | 0.0325 | 0.3768 |
| GO:0002700 | regulation of production of molecular mediator of immune response | 3 | 48 | 48 | 6.25 | 100.00 | 2.6721 | 0.0370 | 0.4066 |
| GO:0006793 | phosphorus metabolic process | 23 | 891 | 903 | 2.58 | 98.67 | 2.6359 | 0.0085 | 0.1730 |
| GO:0006796 | phosphate metabolic process | 23 | 891 | 903 | 2.58 | 98.67 | 2.6359 | 0.0085 | 0.1730 |
| GO:0040029 | regulation of gene expression, epigenetic | 4 | 77 | 80 | 5.19 | 96.25 | 2.6305 | 0.0215 | 0.2951 |
| GO:0002576 | platelet degranulation | 4 | 77 | 79 | 5.19 | 97.47 | 2.6305 | 0.0240 | 0.3169 |
| GO:0002699 | positive regulation of immune effector process | 4 | 77 | 78 | 5.19 | 98.72 | 2.6305 | 0.0250 | 0.3270 |
| GO:0031098 | stress-activated protein kinase signaling cascade | 4 | 77 | 77 | 5.19 | 100.00 | 2.6305 | 0.0300 | 0.3577 |
| GO:0051403 | stress-activated MAPK cascade | 3 | 49 | 49 | 6.12 | 100.00 | 2.6270 | 0.0365 | 0.4046 |
| GO:0033036 | macromolecule localization | 7 | 179 | 179 | 3.91 | 100.00 | 2.6143 | 0.0125 | 0.2308 |
| GO:0052548 | regulation of endopeptidase activity | 7 | 179 | 183 | 3.91 | 97.81 | 2.6143 | 0.0200 | 0.2823 |
| GO:0051649 | establishment of localization in cell | 25 | 996 | 1007 | 2.51 | 98.91 | 2.6068 | 0.0080 | 0.1662 |
| GO:0044419 | interspecies interaction between organisms | 11 | 340 | 340 | 3.24 | 100.00 | 2.5946 | 0.0090 | 0.1813 |
| GO:0071824 | protein-DNA complex subunit organization | 5 | 110 | 115 | 4.55 | 95.65 | 2.5899 | 0.0295 | 0.3545 |
| GO:0006914 | autophagy | 3 | 50 | 51 | 6.00 | 98.04 | 2.5830 | 0.0410 | 0.4315 |
| GO:0046486 | glycerolipid metabolic process | 7 | 181 | 186 | 3.87 | 97.31 | 2.5814 | 0.0150 | 0.2378 |
| GO:0009746 | response to hexose stimulus | 4 | 79 | 79 | 5.06 | 100.00 | 2.5691 | 0.0245 | 0.3216 |
| GO:0007611 | learning or memory | 5 | 111 | 111 | 4.50 | 100.00 | 2.5665 | 0.0170 | 0.2544 |
| GO:0070201 | regulation of establishment of protein localization | 7 | 182 | 184 | 3.85 | 98.91 | 2.5650 | 0.0185 | 0.2687 |
| GO:0006974 | response to DNA damage stimulus | 14 | 477 | 485 | 2.94 | 98.35 | 2.5433 | 0.0110 | 0.2130 |
| GO:0001764 | neuron migration | 3 | 51 | 51 | 5.88 | 100.00 | 2.5402 | 0.0410 | 0.4315 |
| GO:0033043 | regulation of organelle organization | 10 | 303 | 306 | 3.30 | 99.02 | 2.5398 | 0.0135 | 0.2308 |
| GO:0034284 | response to monosaccharide stimulus | 4 | 80 | 80 | 5.00 | 100.00 | 2.5391 | 0.0255 | 0.3313 |
| GO:0030099 | myeloid cell differentiation | 4 | 80 | 80 | 5.00 | 100.00 | 2.5391 | 0.0335 | 0.3852 |
| GO:0034762 | regulation of transmembrane transport | 6 | 147 | 147 | 4.08 | 100.00 | 2.5367 | 0.0150 | 0.2378 |
| GO:0006874 | cellular calcium ion homeostasis | 7 | 184 | 185 | 3.80 | 99.46 | 2.5328 | 0.0175 | 0.2577 |
| GO:0052547 | regulation of peptidase activity | 7 | 186 | 190 | 3.76 | 97.89 | 2.5009 | 0.0220 | 0.3001 |
| GO:0044238 | primary metabolic process | 110 | 5952 | 6073 | 1.85 | 98.01 | 2.5000 | 0.0275 | 0.3416 |
| GO:0033044 | regulation of chromosome organization | 3 | 52 | 52 | 5.77 | 100.00 | 2.4984 | 0.0345 | 0.3935 |
| GO:0043393 | regulation of protein binding | 3 | 52 | 52 | 5.77 | 100.00 | 2.4984 | 0.0350 | 0.3962 |
| GO:0006944 | cellular membrane fusion | 3 | 52 | 53 | 5.77 | 98.11 | 2.4984 | 0.0385 | 0.4181 |
| GO:0046903 | secretion | 12 | 394 | 399 | 3.05 | 98.75 | 2.4870 | 0.0110 | 0.2130 |
| GO:0043408 | regulation of MAPKKK cascade | 7 | 187 | 189 | 3.74 | 98.94 | 2.4851 | 0.0235 | 0.3129 |
| GO:0030168 | platelet activation | 8 | 226 | 229 | 3.54 | 98.69 | 2.4840 | 0.0165 | 0.2483 |
| GO:0043280 | positive regulation of caspase activity | 4 | 82 | 82 | 4.88 | 100.00 | 2.4805 | 0.0315 | 0.3689 |
| GO:0048589 | developmental growth | 5 | 115 | 115 | 4.35 | 100.00 | 2.4751 | 0.0185 | 0.2687 |
| GO:0009653 | anatomical structure morphogenesis | 22 | 870 | 877 | 2.53 | 99.20 | 2.4730 | 0.0150 | 0.2378 |
| GO:0048511 | rhythmic process | 6 | 151 | 153 | 3.97 | 98.69 | 2.4625 | 0.0235 | 0.3129 |
| GO:0051253 | negative regulation of RNA metabolic process | 11 | 353 | 354 | 3.12 | 99.72 | 2.4603 | 0.0120 | 0.2270 |
| GO:0043244 | regulation of protein complex disassembly | 3 | 53 | 53 | 5.66 | 100.00 | 2.4577 | 0.0380 | 0.4150 |
| GO:0045732 | positive regulation of protein catabolic process | 3 | 53 | 54 | 5.66 | 98.15 | 2.4577 | 0.0410 | 0.4315 |
| GO:0048520 | positive regulation of behavior | 3 | 53 | 53 | 5.66 | 100.00 | 2.4577 | 0.0455 | 0.4538 |
| GO:0006730 | one-carbon metabolic process | 6 | 152 | 156 | 3.95 | 97.44 | 2.4443 | 0.0205 | 0.2853 |
| GO:0031334 | positive regulation of protein complex assembly | 3 | 54 | 55 | 5.56 | 98.18 | 2.4179 | 0.0355 | 0.3990 |
| GO:0006606 | protein import into nucleus | 3 | 54 | 54 | 5.56 | 100.00 | 2.4179 | 0.0360 | 0.4017 |
| GO:0070848 | response to growth factor stimulus | 3 | 54 | 54 | 5.56 | 100.00 | 2.4179 | 0.0395 | 0.4247 |
| GO:0042594 | response to starvation | 3 | 54 | 54 | 5.56 | 100.00 | 2.4179 | 0.0405 | 0.4291 |
| GO:0061025 | membrane fusion | 3 | 54 | 55 | 5.56 | 98.18 | 2.4179 | 0.0435 | 0.4466 |
| GO:0043254 | regulation of protein complex assembly | 5 | 118 | 120 | 4.24 | 98.33 | 2.4092 | 0.0265 | 0.3361 |
| GO:0009890 | negative regulation of biosynthetic process | 14 | 494 | 495 | 2.83 | 99.80 | 2.4038 | 0.0205 | 0.2853 |
| GO:0032269 | negative regulation of cellular protein metabolic process | 8 | 232 | 234 | 3.45 | 99.15 | 2.4027 | 0.0200 | 0.2823 |
| GO:0033273 | response to vitamin | 5 | 119 | 120 | 4.20 | 99.17 | 2.3876 | 0.0320 | 0.3732 |
| GO:0007162 | negative regulation of cell adhesion | 3 | 55 | 55 | 5.45 | 100.00 | 2.3791 | 0.0305 | 0.3615 |
| GO:0045834 | positive regulation of lipid metabolic process | 3 | 55 | 56 | 5.45 | 98.21 | 2.3791 | 0.0415 | 0.4341 |
| GO:0019932 | second-messenger-mediated signaling | 9 | 276 | 277 | 3.26 | 99.64 | 2.3681 | 0.0210 | 0.2905 |
| GO:0045934 | negative regulation of nucleobase, nucleoside, nucleotide and nucleic acid metabolic process | 12 | 408 | 409 | 2.94 | 99.76 | 2.3576 | 0.0145 | 0.2330 |
| GO:0043170 | macromolecule metabolic process | 87 | 4602 | 4704 | 1.89 | 97.83 | 2.3507 | 0.0400 | 0.4268 |
| GO:0050890 | cognition | 5 | 121 | 121 | 4.13 | 100.00 | 2.3453 | 0.0220 | 0.3001 |
| GO:0051341 | regulation of oxidoreductase activity | 3 | 56 | 56 | 5.36 | 100.00 | 2.3412 | 0.0460 | 0.4565 |
| GO:0043434 | response to peptide hormone stimulus | 10 | 324 | 325 | 3.09 | 99.69 | 2.3110 | 0.0190 | 0.2740 |
| GO:0051170 | nuclear import | 3 | 57 | 57 | 5.26 | 100.00 | 2.3041 | 0.0395 | 0.4247 |
| GO:0001101 | response to acid | 3 | 57 | 58 | 5.26 | 98.28 | 2.3041 | 0.0480 | 0.4663 |
| GO:0001568 | blood vessel development | 3 | 57 | 57 | 5.26 | 100.00 | 2.3041 | 0.0495 | 0.4762 |
| GO:0019637 | organophosphate metabolic process | 7 | 200 | 206 | 3.50 | 97.09 | 2.2888 | 0.0240 | 0.3169 |
| GO:0007050 | cell cycle arrest | 5 | 124 | 124 | 4.03 | 100.00 | 2.2832 | 0.0300 | 0.3577 |
| GO:0051172 | negative regulation of nitrogen compound metabolic process | 12 | 417 | 418 | 2.88 | 99.76 | 2.2771 | 0.0175 | 0.2577 |
| GO:0007623 | circadian rhythm | 3 | 58 | 58 | 5.17 | 100.00 | 2.2678 | 0.0475 | 0.4644 |
| GO:0051480 | cytosolic calcium ion homeostasis | 5 | 125 | 126 | 4.00 | 99.21 | 2.2630 | 0.0295 | 0.3545 |
| GO:0032940 | secretion by cell | 9 | 286 | 290 | 3.15 | 98.62 | 2.2528 | 0.0250 | 0.3270 |
| GO:0046907 | intracellular transport | 18 | 708 | 715 | 2.54 | 99.02 | 2.2501 | 0.0170 | 0.2544 |
| GO:0043123 | positive regulation of I-kappaB kinase/NF-kappaB cascade | 5 | 126 | 126 | 3.97 | 100.00 | 2.2429 | 0.0370 | 0.4066 |
| GO:0044262 | cellular carbohydrate metabolic process | 12 | 421 | 426 | 2.85 | 98.83 | 2.2420 | 0.0230 | 0.3089 |
| GO:0051707 | response to other organism | 9 | 287 | 293 | 3.14 | 97.95 | 2.2415 | 0.0225 | 0.3049 |
| GO:0071705 | nitrogen compound transport | 6 | 164 | 165 | 3.66 | 99.39 | 2.2367 | 0.0285 | 0.3469 |
| GO:0003012 | muscle system process | 6 | 165 | 166 | 3.64 | 99.40 | 2.2203 | 0.0330 | 0.3810 |
| GO:0046942 | carboxylic acid transport | 6 | 165 | 165 | 3.64 | 100.00 | 2.2203 | 0.0330 | 0.3810 |
| GO:0071842 | cellular component organization at cellular level | 44 | 2114 | 2136 | 2.08 | 98.97 | 2.2198 | 0.0380 | 0.4150 |
| GO:0010952 | positive regulation of peptidase activity | 4 | 92 | 92 | 4.35 | 100.00 | 2.2123 | 0.0435 | 0.4466 |
| GO:0007178 | transmembrane receptor protein serine/threonine kinase signaling pathway | 4 | 92 | 92 | 4.35 | 100.00 | 2.2123 | 0.0470 | 0.4627 |
| GO:0051223 | regulation of protein transport | 6 | 166 | 168 | 3.61 | 98.81 | 2.2039 | 0.0345 | 0.3935 |
| GO:0048858 | cell projection morphogenesis | 5 | 128 | 128 | 3.91 | 100.00 | 2.2034 | 0.0310 | 0.3668 |
| GO:0034641 | cellular nitrogen compound metabolic process | 58 | 2927 | 2966 | 1.98 | 98.69 | 2.2021 | 0.0445 | 0.4499 |
| GO:0015849 | organic acid transport | 6 | 167 | 167 | 3.59 | 100.00 | 2.1877 | 0.0340 | 0.3893 |
| GO:0006633 | fatty acid biosynthetic process | 4 | 93 | 93 | 4.30 | 100.00 | 2.1875 | 0.0435 | 0.4466 |
| GO:0043414 | macromolecule methylation | 4 | 93 | 95 | 4.30 | 97.89 | 2.1875 | 0.0455 | 0.4538 |
| GO:0031327 | negative regulation of cellular biosynthetic process | 13 | 477 | 478 | 2.73 | 99.79 | 2.1647 | 0.0305 | 0.3615 |
| GO:0032870 | cellular response to hormone stimulus | 9 | 295 | 297 | 3.05 | 99.33 | 2.1530 | 0.0255 | 0.3313 |
| GO:0015837 | amine transport | 5 | 131 | 131 | 3.82 | 100.00 | 2.1454 | 0.0360 | 0.4017 |
| GO:0007610 | behavior | 9 | 296 | 296 | 3.04 | 100.00 | 2.1421 | 0.0260 | 0.3350 |
| GO:0045892 | negative regulation of transcription, DNA-dependent | 10 | 341 | 342 | 2.93 | 99.71 | 2.1380 | 0.0275 | 0.3416 |
| GO:0051046 | regulation of secretion | 9 | 297 | 301 | 3.03 | 98.67 | 2.1312 | 0.0285 | 0.3469 |
| GO:0032990 | cell part morphogenesis | 5 | 132 | 132 | 3.79 | 100.00 | 2.1265 | 0.0350 | 0.3962 |
| GO:0071841 | cellular component organization or biogenesis at cellular level | 44 | 2145 | 2167 | 2.05 | 98.98 | 2.1169 | 0.0480 | 0.4663 |
| GO:0005975 | carbohydrate metabolic process | 15 | 581 | 590 | 2.58 | 98.47 | 2.1093 | 0.0355 | 0.3990 |
| GO:0048011 | nerve growth factor receptor signaling pathway | 7 | 213 | 213 | 3.29 | 100.00 | 2.1071 | 0.0360 | 0.4017 |
| GO:0009743 | response to carbohydrate stimulus | 4 | 97 | 97 | 4.12 | 100.00 | 2.0915 | 0.0470 | 0.4627 |
| GO:0009058 | biosynthetic process | 44 | 2153 | 2181 | 2.04 | 98.72 | 2.0906 | 0.0425 | 0.4399 |
| GO:0051052 | regulation of DNA metabolic process | 5 | 134 | 135 | 3.73 | 99.26 | 2.0890 | 0.0360 | 0.4017 |
| GO:0034329 | cell junction assembly | 5 | 134 | 134 | 3.73 | 100.00 | 2.0890 | 0.0475 | 0.4644 |
| GO:0051248 | negative regulation of protein metabolic process | 8 | 258 | 260 | 3.10 | 99.23 | 2.0771 | 0.0290 | 0.3512 |
| GO:0010720 | positive regulation of cell development | 4 | 99 | 99 | 4.04 | 100.00 | 2.0453 | 0.0470 | 0.4627 |
| GO:0006631 | fatty acid metabolic process | 7 | 218 | 218 | 3.21 | 100.00 | 2.0406 | 0.0410 | 0.4315 |
| GO:0043122 | regulation of I-kappaB kinase/NF-kappaB cascade | 5 | 138 | 138 | 3.62 | 100.00 | 2.0162 | 0.0490 | 0.4734 |
| GO:0010558 | negative regulation of macromolecule biosynthetic process | 12 | 448 | 449 | 2.68 | 99.78 | 2.0142 | 0.0430 | 0.4435 |
|  |  |  |  |  |  |  |  |  |  |
| Database version = EnsMart62Plus | |  |  |  |  |  |  |  |  |
| Based on ontology from 6/14/2011 | |  |  |  |  |  |  |  |  |
| Database: Based on OBO-Database version: 6/14/2011 | |  |  |  |  |  |  |  |  |
| Filtering based on the "Number Changed" (>2), "Z Score"(>1.96), and "PermuteP"(<0.05). | | | | |  |  |  |  |  |
| The Z score is based on an N of 17158 and a R of 262 distinct genes in the GO. | | | |  |  |  |  |  |  |
